# What patient-reported outcome measures may be suitable for research involving older adults with frailty? A scoping review

**DOI:** 10.1007/s41999-024-00964-5

**Published:** 2024-03-26

**Authors:** S. O. Long, S. V. Hope

**Affiliations:** 1 University of Exeter, Exeter, UK; 2 Royal Devon University Healthcare NHS Foundation Trust, Exeter, UK

**Keywords:** Frailty, Patient-reported outcome measures, Outcomes, Quality of life, Review

## Abstract

**Introduction:**

The need to develop and evaluate frailty-related interventions is increasingly important, and inclusion of patient-reported outcomes is vital. Patient-reported outcomes can be defined as measures of health, quality of life or functional status reported directly by patients with no clinician interpretation. Numerous validated questionnaires can thus be considered patient-reported outcome measures (PROMs). This review aimed to identify existing PROMs currently used in quantitative research that may be suitable for older people with frailty.

**Method:**

PubMed and Cochrane were searched up to 24/11/22. Inclusion criteria were quantitative studies, use of a PROM, and either measurement of frailty or inclusion of older adult participants. Criteria were created to distinguish PROMs from questionnaire-based clinical assessments. 197 papers were screened. PROMs were categorized according to the domain assessed, as derived from a published consensus ‘Standard Set of Health Outcome Measures for Older People’.

**Results:**

88 studies were included. 112 unique PROMs were used 289 times, most frequently the SF-36 (*n* = 21), EQ-5D (*n* = 21) and Barthel Index (*n* = 14). The most frequently assessed outcome domains included Mood and Emotional Health and Activities of Daily Living, with fewer assessments of Participation in Decision-Making and Carer Burden.

**Conclusions:**

PROM usage in frailty research is highly heterogeneous. Frequently used PROMs omit important outcomes identified by older adults. Further research should evaluate the importance of specific outcomes and identify PROMs relevant to people at different stages of frailty. Consistent and appropriate PROM use in frailty research would facilitate more effective comparisons and meaningful evaluation of frailty interventions.

**Supplementary Information:**

The online version contains supplementary material available at 10.1007/s41999-024-00964-5.

## Key summary points

**Aim:**

Identify and collate existing Patient-Reported Outcome Measures (PROMs) that may be suitable for evaluating the “success” of frailty interventions.

**Findings:**

PROMs are inconsistently used across research with older adults, including people living with frailty. The PROMs that are used may not be the most suitable measures of outcomes that matter to this population.

**Message:**

Research on what matters to people living with frailty and evaluation of PROMs is needed to create consistency in PROM usage and facilitate comparison across studies.

**Supplementary Information:**

The online version contains supplementary material available at 10.1007/s41999-024-00964-5.

## Introduction

### Frailty and interventions

Frailty is a state of reduced physiological reserve, whereby people are vulnerable to adverse outcomes following seemingly trivial stressors [1]. While separate from healthy ageing, frailty risk increases with age; prevalence is 10% for people aged over 65 [2] and 25–50% over 85 [3]. People living with frailty are at increased risk of falls [4], disability [5], hospitalisation [6] and death [7]. As societal life expectancy increases, it is essential to systematically identify and monitor frailty in individuals to offer optimal support, and design and implement interventions to prevent, slow, and even reverse frailty [2].

Frailty ‘interventions’ can be highly varied, from multidisciplinary-based individualised comprehensive geriatric assessment (CGA) approaches to public health-based interventions [8, 9]. The former entails identifying and addressing individuals’ needs with specific support (including exercise programmes, nutritional workshops, medication reviews, and referrals to other professionals).

### Outcome measures

Given the heterogeneity of individual ageing, deciding what outcomes make a frailty intervention “successful” is challenging. Classically, macro-level clinical outcomes including mortality [10], hospitalisation [10], institutionalisation [11] and changes in frailty [12] have been used to measure intervention “success”. Due to the nature of frailty, these may not always be suitable measures, e.g., in severe frailty, adherence to preferred place of death and reducing distressing symptoms may be more relevant than reducing mortality per se [13]. Clinical measures relating to underlying conditions can also be outcome measures, as frailty can be driven by conditions such as osteoarthritis, hypertension, depression and diabetes mellitus [14]. Treatment of such conditions can thus address drivers of frailty. An individual may become more active due to improved joint function or diabetes control, which could contribute to reversing frailty.

Similarly, in clinical decision-making, there is often a balance between prioritising survival and minimising disability [15]. Surgical interventions are often advised against when discussing with individuals with frailty due to the increased mortality risk [16]. However, this may overlook evidence that, for example, patients with frailty who undergo aortic valve replacement [17, 18] or spinal surgery [19] may experience increased quality of life post-operatively. As higher frailty scores were associated with lower baseline quality-of-life scores, people living with more severe frailty often had more to gain from such surgical interventions. Weighing increased risks against possible improvement in quality of life is clearly complex [20].

Systematic assessment of clinical and patient-reported outcomes before and after such interventions, or non-interventions, is thus vital to inform future discussions and evidence-based practice. Researchers and clinicians are increasingly recognising the importance of patient-reported outcomes (PROs), defined as outcomes relating to patients’ health, quality of life or functional status which are reported directly by patients with no interpretation by clinicians [21]. PROs could identify potentially conflicting priorities between traditional research outcomes and individuals, such as the previous example of priorities in severe frailty.

What is important to older people, or ‘what outcomes matter’, has been investigated by various research strategies. Qualitative interviews with older adults in 2006 suggested that participation in activities, good health, and safe home and surroundings influenced quality of life [22]. Quality of life itself was conceptualised as attachment, role, enjoyment, security, and control. This was supported by a meta-synthesis of research involving patients over 80 who received ambulatory care, which found three wishes that mattered: feeling safe, feeling like a meaningful human being, and maintaining control and independence [23]. The ‘What Matters Most—Structured Tool’ has been developed to help older adults express their values and prioritise outcomes that matter to them [24].

Less research has specifically investigated outcomes relevant to people living with frailty. One study identified PROs in people with frailty for evaluating transitional care [25]. For a palliative care intervention with older people with frailty, increased security in care and fewer unmet needs were identified as important outcomes [26]. However, PROs are inconsistently assessed in other relevant scenarios, such as surgery [27]. Two systematic reviews addressing aspects of outcomes used in frailty interventions found that outcomes used varied significantly across studies, and it is also clear that many of the included studies omitted PROMs [28, 29]. Neither of these systematic reviews focused specifically on PROMs used in frailty research however, which is the focus of this review.

### Standard outcome measures

Standard sets of outcome measures are vital in research [30], though geriatrics research has been relatively slow to adopt this approach. Variability of outcomes used in geriatrics research led in 2008 to the development of a 25-item Geriatric Minimum Data Set (GMDS-25) by researchers across Europe, designed to be used in all clinical trials involving older adults, and included PROMs such as the EQ-5D [30]. In the Netherlands, researchers developed The Older Persons and Informal Caregivers Survey Minimum Dataset (TOPICS-MDS), aiming for all Dutch protocols to adopt standardised measures [31].

An International Consortium for Health Outcome Measures (ICHOM) working group investigated outcomes that matter to older adults using a modified Delphi technique, literature reviews, surveys and data from focus groups including older people. These were developed into a ‘Standard Set of Health Outcome Measures for Older Persons’ [32]. The outcomes included minimising falls, loneliness, pain and carer burden, and promoting participation in decision-making. The findings were developed into the ICHOM’s Older Person Reference Guide, published in 2018 [33]. The recommended measures were selected for practical reasons, such as being publicly available/not behind a paywall, and using as few questionnaires as possible to minimise complexity and maximise outcomes measured. This resource seems highly valuable and thus far its uptake has been surprisingly low.

With all attempts at collating standard sets of measures, the suggested (often generic) tools may not match older adults’ preferences on which instruments best measure outcomes that matter to them. Other study design aspects will also affect choice of PROMs used (e.g., intervention type, time constraints, study duration). Given the heterogeneity of ageing and the likelihood of changing priorities over time, it seems likely that these outcomes may not reflect preferences at different degrees of frailty.

This review aimed to identify and collate existing PROMs that may be suitable for evaluating the “success” of frailty interventions, with the research question: What existing PROMs are used in research and may be suitable for older people with frailty? To the authors’ knowledge this is the first review to aim to synthesise the instruments used to measure any patient-reported outcomes (not just quality-of-life instruments). The PROMs identified will then in subsequent research be evaluated for use with older people living with frailty.

## Methods

This report aimed to provide a non-exhaustive but timely review to inform further research on PROMs for frailty interventions. A scoping review approach (compliant with the PRISMA Scoping Review checklist) facilitated the identification and mapping of available PROMs currently used in research [34].

### Search strategy

Reports were identified in October–November 2022 by searching Cochrane and PubMed. Searches used combinations of the following terms: frailty, frailty intervention, outcome, patient-reported outcome measures, patient-reported outcome. The search strategy is available in Appendix 1. One reviewer retrieved and assessed records. The review was not registered, and a protocol has not been published.

Texts were screened in full because of the need to screen PROMs to assess suitability for inclusion. Where PROMs were irretrievable, authors were contacted if possible.

The search was ended on 24th November 2022, when it was agreed that a sufficient number of candidate PROMs (*n* = 112) had been retrieved to meet the aims of this review.

### Inclusion criteria

The criteria were that studies must have:

1. Been an experimental or observational quantitative study.
2. Included some assessment of or screening for frailty, or older adults as participants.
3. Used a PROM that is not disease-specific.

#### Study criteria

Only records written in English were included. Experimental and observational studies were included, as this review focused on PROMs used rather than study results.

Records were excluded if they were not quantitative (e.g., reviews or qualitative research), though relevant references were searched. Abstracts, protocols and trial registrations were included if they specified which PROMs they would use and were not superseded by subsequent publications.

Where multiple records were identified for the same study (i.e., the same participant population underwent the same procedure), each report that described PROMs used was included and grouped as one study. Multiple reports from the same study that did not provide new information about PROM usage were excluded.

#### Participant criteria

Records were included if they assessed or screened for frailty, or they specifically included older adults. This broader search strategy was adopted to identify as many PROMs relevant to frailty as possible, as older trials predated modern frailty assessments but used relevant PROMs.

The authors recognize that not all older people live with frailty. However, a large proportion of people who live with frailty are of an older age. Frailty can be considered as a spectrum from robustness to severe frailty, and older adults may be assessed as being on different points of this spectrum at different points in time. Since this review aimed to identify potentially suitable measures for older people with frailty, instruments that were used in research involving older adults were also considered to be relevant for subsequent evaluation.

#### PROMs criteria

Only PROMs considered potentially relevant for evaluating frailty interventions were included. This excluded disease-specific PROMs (e.g., cancer, heart failure) but included PROMs for chronic illness or end-of-life care. Clinical outcomes (e.g., hospitalisation, mortality) and patient-reported experience measures (PREMs) were excluded.

Clinical assessments were excluded. However, distinguishing questionnaire-based clinical assessments from PROMs can be challenging. To the authors’ knowledge there are no formal criteria to categorize instruments as PROMs. The following criteria were, therefore, developed to include measures as PROMs:

1. The instrument should be from the **patient’s perspective**.
  It should not be technical. A patient with capacity should be able to understand the items and self-report all answers independently, even if they need assistance to physically complete the questionnaire. If one or more items required clinician expertise, this was not included as a PROM.
  At least some items should provide insight into the patient’s perspective which could not be obtained from objective clinical assessment/medical records alone.
2. The instrument could be used to describe a patient’s **quality of life** or their **state of living**.
  Instruments should not only be used to screen for, assess or diagnose a medical condition.
  Answers should not be purely clinical (e.g., medical history).

Measures that did not meet one or more of these criteria were categorized as clinical assessments and were excluded. Instruments that remained ambiguous after applying these criteria were screened by a second reviewer and both reviewers decided whether to include or exclude the instrument. Inclusion decisions were made at the level of the entire instrument rather than individual items, due to the large numbers of instruments identified. In this way, even if a specific item was patient reported, it was only included if the entire measure was assessed to meet the above criteria.

As a consequence, frailty assessments were excluded from the counts of PROMs. For example, the Fried Frailty phenotype [35] includes items such as, ‘In the last year, how often did you feel that everything you did was an effort?’ However, the frailty assessment also includes physical assessments of slowness and weakness, which count as clinical assessments.

### Data analysis

PROMs were categorised where possible using the ICHOM’s ‘Standard Set of Health Outcome Measures for Older Persons’ outcomes: Activities of Daily Living, Pain, Mood and Emotional Health, Participation in Decision-Making, Loneliness and Isolation, and Carer Burden. The number of PROMs in each domain and number of usages of each PROM were tabulated.

PROMs not fitting into the ICHOM’s outcome categories have been presented in categories that best describe their area of measurement. PROMs that measure more than one domain, like the 36-Item Short Form Health Survey (SF-36), were categorized in the domain of Health-Related Quality of Life.

Shortened versions of PROMs were reported as one instrument. For example, the 12-Item and 8-Item Short Form Health Survey (SF-12 and SF-8), which are abbreviated versions of the SF-36, were reported as one instrument.

PROMs incorporating the same items but different scoring systems, like the SF-36 and Veterans RAND 36-Item Health Survey, were counted separately.

Partial usages of shorter instruments (use of some but not all items) were counted as one usage. Partial usages of longer instruments with distinct subsections, such as the iMTA Valuation of Informal Care Questionnaire, are presented separately. Usages of the same instrument for different populations (e.g., Hospital Anxiety and Depression Scale for patients and carers) were counted as one instrument with multiple usages.

Unvalidated ad-hoc items have been included and described according to their assessment domain. For example, use of an unvalidated questionnaire on patients’ physical symptoms has been categorised as “Ad-hoc Symptoms Questionnaire”.

PROMs were excluded if they were not described in enough detail or could not be retrieved or accessed to assess their suitability.

## Findings

### Study selection

**Fig. 1:**
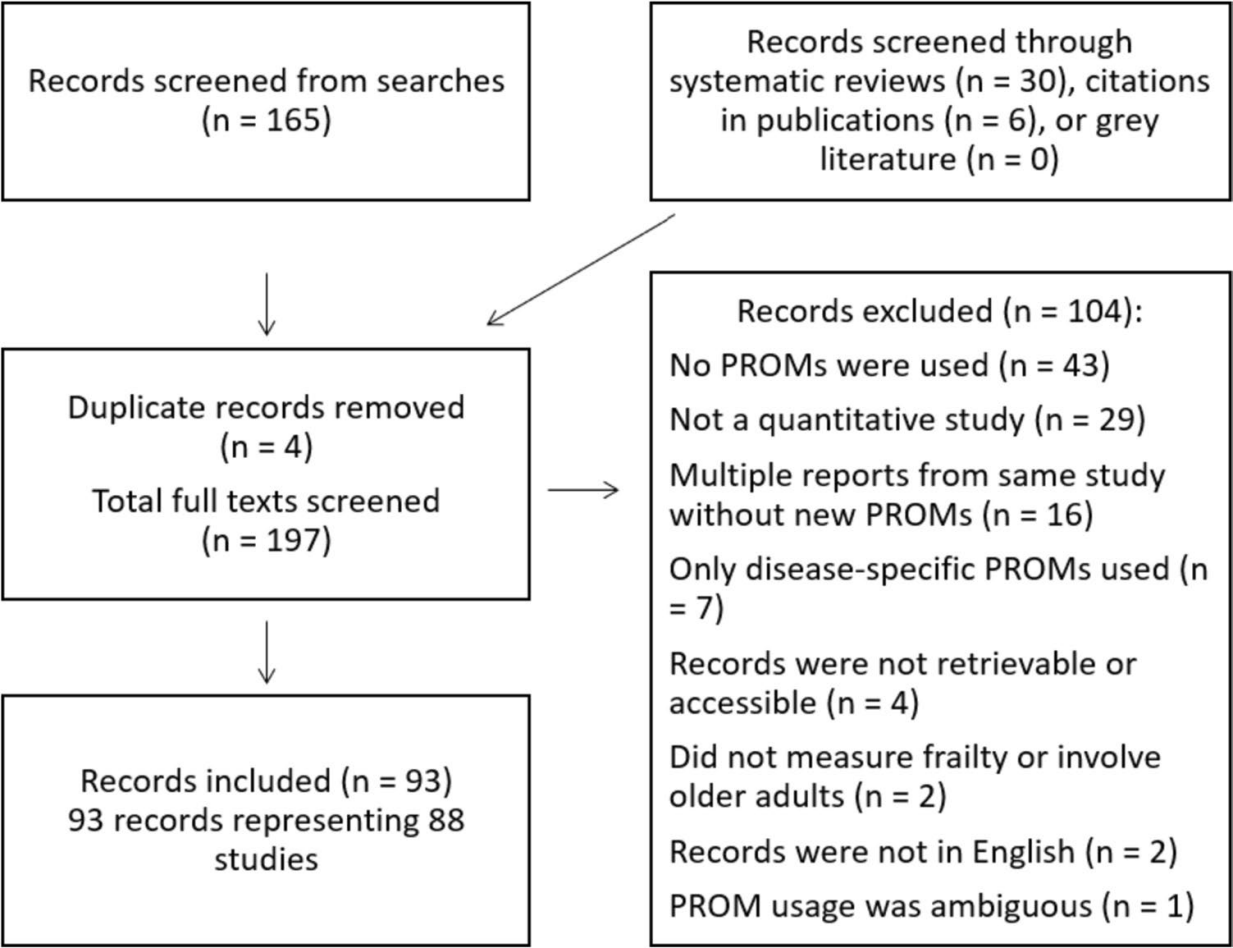
Flowchart describing results of selection process

Figure 1 shows 197 unique records were screened, of which 93 records representing 88 studies were included. 104 records were excluded, most frequently because no PROMs were used (*n* = 43) or the study was not quantitative (*n* = 29). Appendix 2 shows all included studies and the PROMs used.

### PROMs

The aim of the study was to identify a ‘shortlist’ of Patient-Reported Outcome Measures (PROMs) which are consistently used across studies to measure Patient-Reported Outcomes (PROs). PROMs were found to be inconsistently included in research, and no definitive shortlist of PROMs emerged. 112 unique PROMs were used 289 times. Appendix 3 shows all identified PROMs, and Appendix 4 shows examples of scales identified. References for all studies and original references for PROMs are in Appendix 5.

#### Selection of outcome measures

Appendix 2 described the interventions used in each study (if applicable), alongside the PROMs used. Few studies provided details on how they selected instruments to measure outcomes (given that a variety of instruments can measure similar outcomes). One study (S6) detailed that PROMs were selected from a literature review and discussions with stakeholders. Other studies selected generic instruments that were validated for use in disease-specific patient populations included in those studies, such as patients living with cirrhosis (S9), systemic lupus erythematosus (S11), and heart failure (S14).

Only two included studies (S35, S67) explicitly cited the ICHOM’s guidance when selecting PROMs. One study used the TOPICS short form questionnaire (S23) (though scores were only reported as part of a frailty index).

Due to the wide variety of interventions (e.g., surgery, pharmaceutical interventions and multimodal frailty interventions), it is difficult to make generalisations about the relationship between type of interventions and the outcomes selected. PROMs usually seemed to be thematically related to the intervention used in a study, for example, a PROM relating to evaluating palliative care following a palliative care intervention (S43), or relating to pain experience following a pain intervention (S26). Nutritional interventions often clinically assessed the nutritional status of participants, but these measures were excluded due to meeting the criteria of questionnaire-based clinical assessments rather than PROMs.

Some studies used PROMs that were less directly related to the intervention, but were related to higher-level aspiration outcomes such as assessing social support after home-based physiotherapy (S60). More global measures of quality of life were frequently used—and beyond—for example, an aortic valve replacement study using a linear analogue self-assessment scale for spiritual wellbeing (S20).

Despite this, PROMs were used inconsistently even across studies that implemented similar interventions. For example, three studies assessing the effects of prehabilitation for patients undergoing surgery used different PROMs to assess HRQoL and physical activity/activities of daily living (S1, S54, S62). Of five studies assessing exercise interventions, two used different physical activity PROMs (S34, S83), two used different mobility confidence PROMs (S34, S67), and three used different ADL measures (S40, S67, S83), though there was convergence by studies for measures of HRQoL (S66, S67) and mood (S34, S67). In trials investigating pharmaceutical interventions, outcomes that were measured included fatigue and quality of life (S2), two different measures of mood (S58, S64) and measures of ADLs, mobility confidence and sexual quality of life (S58).

#### Frequently used measures

Table 1 shows instruments used four times or more. Citations after each measure name refer to either the development or validation of the measure (as opposed to research studies that merely used this measure). Citations in the last column show whether the instrument has been specifically validated for use in people living with frailty or older adults. Validating an instrument requires assessing its validity (whether the questionnaire measures what it aims to measure) and reliability (whether individuals’ responses remain consistent) [36].

**Table 1:** PROMs with the highest frequency of usage

Measure name	Frequency of usage	Domain	Description	Validation for people with frailty/older people
Short Form Health Survey (SF-36, SF-12 and SF-8) (S126)	21	Health-related quality of life	Assesses eight domains: physical functioning, physical role, pain, general health, vitality, social function, emotional role, and mental health 36-item, 12-item and 8-item versions exist (SF-36, SF-12 and SF-8) Contains same items as Veterans RAND Health Survey but scored differently	Internal consistency and test–retest reliability did not meet standards for clinical application of SF-36 in older people with frailty [37]
EQ-5D (S127)	21	Health-related quality of life	5 items. 3-Level and 5-Level versions exist (EQ-5D-3L and EQ-5D-5L). Contains Visual Analogue Scale for health	Good test–retest reliability, construct validity and responsiveness of EQ-5D-5L in older people with frailty. EQ-5D-3L was more strongly associated with physical limitations and less strongly with aspects beyond physical health [38]
Barthel Index (S94)	14	Activities of daily living	10 items assessing activities of daily living (ADL), with three levels each	A Japanese study found partial correlation between BI and Role Emotional domain of SF-8 in older people using long-term care insurance services [39] (Frailty was not measured in this study.)
Geriatric Depression Scale (GDS) (S143)	12	Mood and emotional health	Assesses symptoms of depression on a 2-point scale. 30-item, 15-item and 5-item versions exist	GDS compared with Research Diagnostic Criteria for Depression and was found to be reliable and valid for elderly populations [S143] (No validation found for older people with frailty specifically.)
Lawton-Brody Instrumental Activities of Daily Living (IADL) Scale (S95)	11	Activities of daily living	Assesses eight activities including using telephone, food preparation and housekeeping. Scoring is different for males and females Same development reference as the Physical Self Maintenance Scale	Katz-15 (modified version of Katz Index of ADL and Lawton–Brody IADL scale) was found to be both internally consistent and strongly associated with quality-of-life measures (SF-36, EQ-5D and EQ-5D + C) for older people living with frailty [40]
Katz Index of Activities of Daily Living (ADL) (S96)	9	Activities of daily living	Assesses six activities of daily living on a 2-point scale, including bathing, feeding and toileting	Katz-15 (modified version of Katz Index of ADL and Lawton–Brody IADL scale) was found to be both internally consistent and strongly associated with quality-of-life measures (SF-36, EQ-5D and EQ-5D + C) for older people living with frailty [40]
Falls Efficacy Scale International (FES-I) (S140)	7	Mobility confidence	16 items on a 4-point scale Assesses fear of falling while performing specific activities	A Taiwanese study found the 7-item FES-I was positively associated with fall history and physical frailty and negatively associated with the SF-8, independent of physical frailty [41]
Center for Epidemiologic Studies Depression (CES-D) Scale (S144)	7	Mood and Emotional Health	20 items assessing symptoms of depression, with four levels each	11-item CES-D scale was suggested to be reliable and valid in homebound medically ill older adults [42] (No validation found for older people with frailty specifically.)
Hospital Anxiety and Depression Scale (HADS) (S116)	6	Mood and emotional health	Seven items on anxiety and seven items on depression on a 4-point scale	HADS is valid instrument with satisfactory internal consistency in Swedish older adults (aged 65–80) [43] (No validation found for older people with frailty specifically.)
Patient-Reported Outcomes Measurement Information System (PROMIS) Physical Function [44]	6	Activities of daily living	Items assess individual’s ability to perform physical tasks in daily life, on a 5-point scale Multiple versions exist, including Physical Function, Physical Function—Upper Extremity, and Physical Function—Mobility	Physical Function-Geriatric Rehabilitation subset of items showed sufficient test–retest reliability, measurement error, and construct validity, but not responsiveness for inpatient geriatric rehabilitation patients [45] (No validation found for older people with frailty specifically.)
Veterans Rand Health Survey (VR-36 and VR-12) (S128)	5	Health-related quality of life	Assesses eight domains, including physical health limitations, mental health, and vitality. 36-item and 12-item versions exist (VR-36 and VR-12) Contains same items as Short Form Health Survey but scored differently	Internal consistency and test–retest reliability did not meet standards for clinical application of SF-36 in older people with frailty [37]. (VR-36 contains same items as SF-36 but scored differently.) Adapted version of VR-12 was found to be valid and reliable in Canadian residents of care homes [46]
Older Americans Resources and Services (OARS) – Activities of Daily Living [47]	4	Activities of daily living	Subsection of the OARS Multidimensional Functional Assessment Questionnaire (MFAQ) Contains seven items on Instrumental Activities of Daily Living (IADL) and eight items on Physical Activities of Daily Living (ADL). 5-item subscale of IADL also exists	Found to be a valid measure when administered to older adults attending emergency department in Canada [48] (No validation found for older people with frailty specifically.)
World Health Organisation Disability Assessment Schedule (WHODAS) [49]	4	Activities of daily living	Assesses six domains, including mobility, self-care and social interaction 36-item and 12-item versions exist (WHODAS-36 and WHODAS-12)	When administered to older adults in Singapore, WHODAS was found to have a high internal consistency and support for the measurement invariance and convergent validity [49] (No validation found for older people with frailty.)

The most frequently used instruments were the SF-36 (and abbreviated versions), which assesses eight domains of health-related quality of life (HRQoL), and the EQ-5D, which assesses five domains of HRQoL and contains a Visual Analogue Scale for health. These were followed by the Barthel Index, measuring 10 activities of daily living (ADLs) including bathing, dressing and feeding.

#### Application of inclusion criteria

Due to the lack of formal criteria, it was difficult to determine the boundary between PROMs and questionnaire-based clinical assessments and tools for clinicians. Measures were assessed using the criteria in the “Methods”, and if excluded, were excluded at the time of data collection. Table 2 shows examples of measures that were included and excluded, and justifications for these decisions.

**Table 2:** Examples of included and excluded measurements with reasons

Examples of excluded measures	Assessment domain	Inclusion decision	Reasons
Karnofsky Performance Scale [50]	Activities of daily living	Exclude	Not patient-reported—individuals would be unable to self-report their own mortality
Self-reported hospitalisation or institutionalisation (e.g., (S1,S7,S9))	Healthcare usage	Exclude	Reports only healthcare usage rather than patients' perceptions, and so does not describe quality of life (some individuals prefer hospitalisation)
Kansas City Cardiomyopathy Questionnaire (KCCQ) [51]	Health-related quality of life	Exclude	Too disease-specific for people living with frailty—not all will have heart failure symptoms
Medication Appropriateness Index [52]	Medication satisfaction/burden	Exclude	Not patient-reported—requires clinician’s technical knowledge
Brief Psychiatric Rating Scale (BPRS) [53]	Mood and emotional health	Exclude	Not patient-reported—patients would be unable to self-rate whether their thoughts are 'realistic'. More of a clinical assessment
Katz Index of Activities of Daily Living (ADL) (S96)	Activities of daily living	Include	Instrument was intended to be completed by observation, but patients frequently self-report answers [54] (though clinician may complete instrument)
Medication Risk Questionnaire (MRQ-10) (S138)	Medication satisfaction/burden	Include	Instrument is technical, but some items provide insight into patient experience that could not be extracted from medical records
Hospital Anxiety and Depression Scale (HADS) (S116)	Mood and emotional heath	Include	Although instrument can screen for anxiety and depression, answers can also describe quality of life. All items are self-reported
Geriatric Pain Measure (S155)	Pain	Include	Pain is a specific symptom, but instrument is sufficiently general to be potentially relevant to older adults living with frailty

#### Ad-hoc measures

There were 21 usages of ad-hoc measures. This includes instruments that were self-designed for particular studies and appear to be unvalidated, measures adapted from validated instruments without evidence of validation of the adapted version, and measures without a referenced instrument. Table 3 shows the frequency of ad-hoc measures by the domain that they measured. The domains most frequently assessed with ad-hoc measures were Activities of Daily Living (ADL) (*n* = 7) and Instrumental Activities of Daily Living (IADL) (*n* = 6).

**Table 3:** Frequency of usage of ad-hoc measures

Ad-hoc measure domain	Frequency of usage
Activities of daily living (ADL)	7
Instrumental activities of daily living (IADL)	6
Health-related quality of life—symptoms	3
Activities of daily living—mobility*	1
Healthcare satisfaction	1
Medication satisfaction/burden	1
Physical activity	1
Social activities	1
Total	21

*This measure was used in combination with a separate Ad-hoc ADL questionnaire and was thus counted separately to the ADL questionnaire

Ad-hoc measures were included in this review, given that some ad-hoc measures included items that may be relevant to older people with frailty with subsequent validation. For example, one ad-hoc measure on mobility included (unusually) an item on driving [55], which may be important to older people with frailty for facilitating other activities.

#### Scales

Definitions of scales were sought to accurately report usages of scales. Supplementary Fig. 1 in Appendix 4 presents examples of rating scales for pain, with reference to definitions of the Likert Scale [56, 57], Verbal Rating Scale (VRS) [56, 57], Visual Analogue Scale (VAS) [21, 58–60], Graphic Rating Scale (GRS) [61] and Numeric Rating Scale (NRS) [60]. Boundaries are not always clear: the Likert Scale can be considered as a bidirectional type of VRS, and VAS can refer to solely the traditional VAS scale, or to the GRS or the linear version of the NRS.

The Linear Analogue Scale (LAS), or Linear Analogue Self-Assessment Scale (LASA), is not shown in Supplementary Fig. 1 in Appendix 4 because of ambiguity in its presentation; historically the LAS referred to the traditional VAS [62], but it is more recently used to refer to the linear version of the NRS [63]. Due to this inconsistency, the LAS has been reported separately to the VAS and NRS.

This review counted all rating scales for a particular outcome as one instrument to avoid inflating the count of PROMs. For example, a pain NRS and a pain VAS were counted as one instrument, referred to as “Pain Scale”. A disaggregated count of outcome by type of scale (as described by authors) is shown in Table 4. This excluded scales used as part of validated questionnaires, such as in the EQ-5D.

**Table 4:** Frequency of usage of types of rating scales

Outcome measurement	Type of rating scale				Total by outcome
	Likert	Visual analogue	Linear analogue	Numeric	
Pain (overall, leg, back, chronic)		4		4	8
Health/physical wellbeing	3		2		5
Quality of life	1	1	2		4
Mental/emotional wellbeing			2		2
Fatigue		1			1
Fear of falling		1			1
Self-rated burden		1			1
Sleep quality				1	1
Social activity			1		1
Spiritual wellbeing			1		1
Total by rating scale	4	8	8	5	
Overall total					25

Rating scales were used most commonly for pain (*n* = 8), followed by health or physical wellbeing (*n* = 5). VAS and LAS were the most frequently used types of rating scale, both used eight times among the included studies. Few studies justified why specific types of scales had been used over others (e.g., why a Likert scale was used instead of a VAS). Not all studies reported the wording used for the instructions or anchors (verbal labels at the scale extremities).

### Outcome domains

There is no definitive list of outcome domains that is mutually exclusive but collectively exhaustive. The authors used the ICHOM domains as a starting point, but some PROMs did not fit in these domains, resulting in judgements by the authors over what additional outcome domains best described these PROMs. For example, the ICHOM recommends measuring Carer Burden. This was interpreted by authors to refer to the burden of caregiving that carers experience. However, many instruments that were completed by carers did not directly assess the pressures of caregiving, and so additional outcomes were created to categorise these instruments.

Table 5 shows a summary of how many instruments were in each outcome domain. The first six domains correspond to the ICHOM’s outcome domains [32]. All domains not included in this standard set are shown below. Table 5 shows that there is considerable variation in the outcomes measured across studies.

**Table 5:** Number of instruments by outcome domain

Outcomes important to older adults (ICHOM)	Number of instruments	Frequency of usage
Activities of daily living	18	76
Mood and emotional health	11	39
Carer burden	6	7
Pain	4	11
Participation in decision-making	3	3
Loneliness and isolation	2	3
Total	44	139

Additional outcome domains	Number of instruments	Frequency of usage
**Physical health**		
Health-related quality of life	15	71
Fatigue	7	8
Physical activity	7	10
Mobility confidence	4	10
Sexual quality of life	3	6
Sleep quality	3	4
Psychological functioning	1	1
Total	40	110
**Healthcare**		
Palliative care and wellbeing	3	4
Healthcare satisfaction	3	5
Medication satisfaction/burden	2	3
Transitional care	2	2
Total	10	14
**Social wellbeing**		
Social activities	3	4
Social support	4	5
Total	7	9
**Carer wellbeing**		
Carer: healthcare satisfaction	2*	2
Carer: mood and emotional health	1*	1
Carer: physical health	1*	1
Carer: autonomy and control	1	1
Carer: work satisfaction	3	3
Total	5**	8
**Other**	6	9
**Overall total of instruments**	112	289

*Includes one instrument already counted in other domains

**Repeated use of instruments counted in other domains have been excluded from the final total

## Discussion

### Multiple PROMs in use

Patient-reported outcomes measures (PROMs) are measures of outcomes including health, quality of life or functional status which are reported directly by patients without clinician interpretation [21]. This review aimed to identify a ‘shortlist’ of questionnaires which could be classed as PROMs that are currently used across quantitative studies of older people or those living with frailty to measure patient-reported outcomes (PROs). The findings were that PROMs were not consistently used across studies, and therefore no shortlist of PROMs could be identified. 112 unique instruments were found to have been used 289 times. The most frequently used included the SF-36 and EQ-5D, both measuring Health-Related Quality of Life (HRQoL), and the Barthel Index, measuring Activities of Daily Living (ADLs).

### Outcome domains that matter to older people

Previous research has investigated outcomes that matter to older adults in ambulatory care [23], identified what quality of life means to older people [64], designed tools to express important outcomes [24], and developed guides to recommend PROMs that measure these outcomes [32, 33], such as the International Consortium for Health Outcome Measures (ICHOM)’s ‘Standard Set of Health Outcome Measures for Older Persons’ [32]. These ICHOM domains were used to categorise PROMs identified in the current study: Activities of Daily Living, Mood and Emotional Health, Carer Burden, Pain, Participation in Decision-Making, and Loneliness and Isolation. 44 tools were found to fit these categories, with the most frequently assessed ICHOM outcome domains being Mood and Emotional Health, and Activities of Daily Living. Infrequently assessed ICHOM domains included Participation in Decision-Making, and Carer Burden.

The review identified over 60 additional questionnaires which fit the definition of PROMs, but did not fit into the ICHOM outcome domains. New domains were, therefore, defined comprising Physical Health, Healthcare domain, Social Wellbeing, and Carer Wellbeing (full list of outcome domains in Table 5). These additional domains are consistent with previous work on outcomes that matter to older people, such as participation in activities, good health, and safe home and surroundings, which were found to influence quality of life [22].

Many of the PROM domains, such as Mood and Emotional Health, Activities of Daily Living, Social Support, and Medication Satisfaction/Burden, overlap with domains that are incorporated in the Comprehensive Geriatric Assessment (CGA), a holistic frailty intervention which is routinely performed by geriatricians [8]. The regular assessment and discussion of these domains in routine clinical care may be one reason why PROMs are not routinely used in clinical geriatrics (in comparison e.g., to surgical specialities), and perhaps geriatrics research. Clearly in research it is important to be able to measure and compare outcomes relevant to the population studied. Well-designed and appropriately used PROMs could also benefit clinical practice, such as highlighting disparity between clinicians’ and patients’ perceptions as discussed below.

It thus seems there are examples of existing PROMs for all the categories of outcomes that have previously been identified as important for older people. However as previously discussed, less research has focused on older people living with frailty, where there could be a wide spectrum of differing priorities, particularly at different stages of frailty. This may explain the relatively high frequency of “ad-hoc” PROMs identified, i.e., study-specific questionnaires or modified versions of other questionnaires. In this review, 21 separate ad-hoc PROMs were identified.

Ad-hoc questionnaires were most frequently used to measure ADLs, despite the identification of 15 validated ADL instruments in this review. There are many reasons why researchers may design their own PROMs, including accessibility of resources (e.g., not behind paywalls) and relevance of instruments to their population (e.g., selecting or designing ADL instruments that suit participants’ mobility). Nevertheless, it is beneficial to use instruments that have been validated for use in older adults, and ideally those living with frailty, to ensure that the data is relevant, accurate and complete.

### Selecting PROMs in research with frail/older people

PROMs are valuable tools to give accurate insight into patients’ priorities, not least because they have demonstrated disparity between clinicians’ and patients’ perceptions. For example, clinicians have been found to overestimate social activity levels and self-reported physical functioning for patients with frailty [65], quality of life for patients with heart failure [66], and underestimate the importance of functional limitations to older people [67]. In care homes, family members and staff have been found to underestimate quality of life for residents with dementia [68].

The significant heterogeneity in PROMs use found in this review is consistent with two previous systematic reviews which found significant heterogeneity in outcomes (not specifically PROMs) used in trials of older people living with frailty [28, 29]. Both reviews concluded that more consistency in the outcomes measured would aid reporting and evaluation of future research studies. The most recent of these systematic reviews (published in 2021 [29]) summarized frailty measures and outcomes reported in trials involving frail older inpatients. Interestingly they observed that the most recently published studies included “relatively narrow, intervention-specific outcomes” as opposed to functional status and quality of life which “may be more meaningful to frail patients and their caregivers”.

Given the holistic nature of geriatrics and recognition of frailty as an overarching condition, this direction of travel seems surprising. Two other systematic reviews which concentrated on quality of life in older adults receiving aged care services [69], and self-reported wellbeing measures in adults [70], similarly concluded there was considerable heterogeneity in instruments used, and that some standardisation is needed in relation to QoL measures in studies with older people [69]. It is important to recognize the benefits and limitations of the scope of assessment of different PROMs. PROMs that measure narrow outcomes (such as the Falls Efficacy Scale International (S140) measuring fear of falling) may not show improvement for unrelated interventions. Furthermore, improvements in a narrow domain may not translate to improvements in global quality of life. On the other hand, PROMs measuring global wellbeing (such as a Linear Analogue Scale for overall Quality of Life) may not reveal what is affecting global wellbeing. It may thus be advantageous to supplement PROMs assessing global wellbeing with more specific PROMs to investigate what is driving any change in overall quality of life. For these reasons, the authors advocate for the use of a selection of PROMs in frailty research, measuring both global wellbeing and specific outcomes that matter to people living with frailty. There is, however, a risk of “questionnaire-fatigue”, adding to the importance of careful consideration of the most relevant and discerning questionnaires to help evaluate interventions in older people living without/with differing degrees of frailty.

Standardizing measures of outcomes that matter to older adults with frailty would thus create consistency in the literature and facilitate the comparison of interventions. This could be achieved by greater dissemination of existing guidelines specifying PROMs to measure outcomes, such as the ‘Standard Set of Health Outcome Measures for Older Persons’ [32]. An alternative approach could be the development of guidelines for selecting PROMs. Such recommendations would not give instructions on which PROMs exactly to use, but could provide a checklist of factors to consider (e.g., validation of the instrument in the patient population, its length, and how the instrument compares to other instruments measuring the same outcome).

It is essential to consider the suitability of PROMs for this population. Although one study found good test–retest reliability, construct validity and responsiveness of EQ-5D-5L in older people with frailty [37], another in people aged over 75 found it to have little reliability and insufficient responsiveness [71]. Internal consistency and test–retest reliability did not meet standards for clinical application of SF-36 in older people with frailty [37]. Yet these were the most frequently used individual PROMs in our survey. Self-completion of the Barthel Index has been found to result in higher scores than actual ADL function in in-patients aged 75 or over [72]. Even factors like instrument layout can cause confusion among older adults, and missing data for the SF-12 was reduced when its layout was changed [73]. In comparison, the Adult Social Care Outcomes Toolkit (ASCOT) was found to have good responsiveness and measures other outcomes that matter to older people [38].

To the authors’ knowledge, no frailty-specific PROMs exist. One measure identified, the Modified Spitzer Quality of Life Index [S89], had been modified to reflect quality of life for individuals with frailty, though was excluded in the counts due to not being a patient-reported tool. However, many pre-existing PROMs measuring HRQoL have been widely used in older populations [69]. Some quality-of-life measures developed since 2000 have been designed specifically for older people, with some convergence identified “towards greater coverage of subjective and person-centred conceptualisations of life quality, and a decrease in focus on physical health” [69].

As the proportion of older adults increases, it is essential to design both interventions for frailty and relevant instruments to assess quality of life and other outcomes important to older people living with different degrees of frailty severity. Further research on what these outcomes are could facilitate the development of a frailty-specific PROM. Alternatively, a “standard set of outcomes that matter to people with frailty” could be developed, while acknowledging that priorities may change with frailty progression.

### PROMs for lesser-addressed domains

Domains assessed as important to older people by the ICHOM but less frequently addressed included Carer Burden, Participation in Decision-Making, and Loneliness and Isolation.

Regarding caregiving burden, the ICHOM recommends the Zarit Caregiver Burden Assessment (S110), found to have been used twice in this review. However, the ICHOM only recommends measuring caregiving burden, and does not propose measures for other outcomes that may matter to carers. For example, an intervention increasing formal care may relieve informal carers of care-giving tasks, but not improve their mental health. This review found that multiple measures used in research with carers did not directly measure this domain, presumably recognising that other outcomes matter to informal carers as well as caregiving burden. For example, the Hospital Anxiety and Depression Scale (S116) was found to have been used once with carers.

For formal caregivers, instruments like the Copenhagen Burnout Inventory (S117) were rarely used. Ensuring healthcare professionals are supported can also improve the QoL of both healthcare professionals and patients; patients have described feeling like a ‘meaningful human being’ after positive experiences with healthcare workers [23].

In relation to participation in decision-making and support for older people, the ICECAP Supportive Care Measure (ICECAP-SCM) (S157) seems useful. It measures outcomes including decision-making opportunities, feeling supported, and reducing suffering, corresponding to outcomes identified in previous research [23], and may be relevant to patients with severe frailty who are receiving palliative care.

### Economic evaluations

Many studies performed economic evaluations of interventions. While these can guide policy decisions, this review focused on PROMs. Economic evaluations often use quality-adjusted life-years (QALYs) [74], where one QALY is one additional year of life in perfect health. QALYs are composite measures of physical health, quality of life, and quantity of life [22], which are all important but conceptually distinct. QALYs can involve PROMs such as the EQ-5D, ASCOT, and the ICEpop CAPability measure for older people (ICECAP-O). PROMs used in this way were counted in this review. However, the QALY itself was not included as a PROM, as this adopts an economic rather than patient perspective (patients cannot report the number of years they survive).

### Strengths and limitations

This review was completed in a short time period to identify potentially relevant PROMs for evaluating frailty interventions. The authors hope that Appendix 2 in particular may be a useful guide to researchers and clinicians when selecting PROMs.

This review is distinct from previous systematic reviews [28, 29] by investigating *instruments* that could be relevant to people with frailty. We feel this review shows a potential lack of inclusion in research studies of outcomes that matter to older people with frailty, and an inconsistency in how patient-reported outcomes are operationalised, resulting in a need for caution when comparing outcomes between studies.

It is acknowledged that this review has limitations. Due to time and funding constraints, records were assessed by one reviewer only, and the review was non-exhaustive. As well as screening papers for inclusion, each instrument identified was also screened in full for inclusion to assess whether all items in the instrument were patient-reported. As a result, the level of detail of reporting on instruments resulted in a compromise on the scale of the search, as large numbers of PROMs were accrued rapidly. Additional problems included inconsistent nomenclature of PROMs and scales, and ad-hoc questionnaires, as described in the “Methods” and “Findings”.

There were two problems when searching the literature with the term ‘frailty’. The first problem is that many papers predated modern frailty assessments, as discussed in the “Methods”, but still used the term ‘frailty’. The second problem is that frailty definitions can vary across studies. Geriatric medicine and research often interprets ‘frailty’ to mean physical frailty such as measured by the Fried Frailty Phenotype [35], where frailty is a potentially reversible condition and not a palliative term. However, frailty is also interpreted as referring to patients living with advancing disease and palliative needs, which may be closer to the Rockwood definition of frailty [75]. Many of the older papers included appeared to use the latter definition implicitly when using terms such as ‘frail’ or ‘frailty’. This inconsistency in terminology adds further complexity to the literature on frailty. As a result, the definition of frailty used by each study (if any) has been documented in Appendix 2. Crucially, frailty severity is not static and so there is not necessarily a binary distinction between suitable PROMs for older people and suitable PROMs for older people with frailty, since older people who may be robust could later be assessed as living with frailty, while others may be able to slow or reverse frailty with targeted interventions. PROMs that were used with older adults could be relevant for evaluating frailty interventions for physical frailty, even if the study that used them did not measure frailty. Therefore, these PROMs still were included in this review.

Future research could include a systematic review on PROMs used in frailty interventions. However, it is also difficult to see how an exhaustive systematic review could be implemented without clear criteria to distinguish PROMs from questionnaire-based clinical assessments. While the same instruments can both describe quality of life and screen for medical problems, these purposes remain distinct and multiple ‘borderline’ instruments were identified. Creating formal criteria to define PROMs could better serve patients in clinical practice, researchers and participants, by ensuring respondents complete questionnaires designed and validated for self-report usage.

More importantly, we would suggest that research should recognise and prioritise measuring outcomes that matter to older people living with different degrees of frailty, and efforts should focus on standardising the use of a smaller number of relevant PROMs, to better assess and compare frailty interventions.

## Conclusion

This review aimed to provide an informative summary of questionnaires which could be classed as PROMs currently used in research with people living with frailty. Usage was found to be highly heterogeneous. The most frequently used may not measure all outcomes that matter to older adults. The authors propose to evaluate identified PROMs in qualitative interviews and explore the importance of outcomes at varying degrees of frailty.

### Supplementary Information

Below is the link to the electronic supplementary material.

Supplementary file1 (DOCX 373 KB)

## Data Availability

Data are available in Appendices 2 and 3 in Supplementary material.
