# Supplementary material for: What patient-reported outcome measures may be suitable for research involving older adults with frailty? A scoping review
Source: Eur Geriatr Med. 2024 Mar 26;15(3):629–44. doi: 10.1007/s41999-024-00964-5 (PMC11329537; doi:10.1007/s41999-024-00964-5)
Supplement: Supplementary file 1 — Supplementary file1 (DOCX 373 KB) [file 41999_2024_964_MOESM1_ESM.docx]

**What patient-reported outcome measures are used in research involving older adults with frailty? A scoping review**

**Authors and Affiliations**

Long SO BA, Graduate Research Associate, University of Exeter, Exeter

Hope SV MBChB PhD FRCP, Honorary Senior Clinical Lecturer, University of Exeter, Exeter; Consultant in Healthcare for Older People and General Internal Medicine, Royal Devon University Healthcare NHS Foundation Trust, Exeter

**Corresponding Author**

Hope SV MBChB PhD FRCP, Honorary Senior Clinical Lecturer, University of Exeter, Exeter; Consultant in Healthcare for Older People and General Internal Medicine, Royal Devon University Healthcare NHS Foundation Trust, Exeter

Email: s.v.hope@exeter.ac.uk

ORCID ID: 0000-0001-7343-0149

**Supplementary Information**

Contents

[Appendix 1 – Search Strategy 3](#_Toc155709866)

[Appendix 2 – Table of included studies and PROMs that each study used 4](#_Toc155709867)

[Appendix 3 – Complete List of Included PROMs 27](#_Toc155709868)

[Appendix 4 – Supplementary Fig. 1 Examples of Types of Rating Scales for Pain 41](#_Toc155709869)

[Appendix 5 – Supplementary Reference List 42](#_Toc155709870)

# Appendix 1 – Search Strategy

**PubMed Search Terms:**

Double quotes were used when searching phrases with more than one word.

(("patient reported outcome measures") AND (frailty))

(("frailty intervention") AND (outcome))

The search was stopped on 18/11/2022.

**Cochrane Search Terms:**

((frailty) AND (patient reported outcome))

The first 56 references were screened for inclusion, until authors agreed on 17/11/2022 that a sufficient number of PROMs (n=112) had been retrieved to meet the aims of the review.

**Additional Records:**

36 records were screened from systematic reviews and citations in publications.

# Appendix 2 – Table of included studies and PROMs that each study used

| **Citation** | **Aim** | **Study Design** | **Intervention** | **Frailty measure (or age group)** | **PROMs used** |
| --- | --- | --- | --- | --- | --- |
| Carli, F, Bousquet-Dion, G, Awasthi, R, et al. (2020) (1) | Investigate effects of multimodal prehabilitation vs postoperative rehabilitation on patients with frailty undergoing resection of colorectal cancer | RCT | Multimodal prehabilitation (exercise, nutrition and psychological interventions) for colorectal cancer surgery | Fried Frailty Index | SF-36 HADS CHAMPS |
| Justice, JN, Nambiar, AM, Tchkonia, T, et al. (2019) (2) | Investigate dasatinib plus quercetin for reducing cellular senescence in patients with idiopathic pulmonary fibrosis | Pilot study | Senolytic drug intervention | Derived frailty index from clinical history | Pittsburgh Fatigability Scale (PFS)  Fatigue Severity Scale (FSS)  Fatigue Scale (Visual Analogue)  Quality of Life Scale (Likert)  Quality of Life Scale (Visual Analogue) |
| Moye, J, Driver, JA, Owsiany, MT, et al. (2022) (3) | Evaluate and refine the What Matters Most – Structured Tool for older adults with multicomplexity | Observational cohort study | Not applicable | FRAIL Scale | MOS Social Support Survey (1 item used) |
| Kusunose, M, Sanda, R, Mori, M, et al. (2021) (4) | Examine associations between frailty and PROs in elderly patients with asthma | Cross-sectional observational study | Not applicable | Kihon Checklist | SF-36 Hyland Scale |
| Frantzen, AT, Eide, LSP, Fridlund, B, et al. 2021 (5) | Determine if frailty changed 6 months after surgical aortic valve replacement in octogenarians. Describe changes in self-rated health for frail patients who underwent SAVR or transcatheter aortic valve implantation | Prospective cohort study | Transcatheter aortic valve implantation (TAVI) and surgical aortic valve replacement (SAVR) | Study of Osteoporotic Fractures Index | SF-12 World Health Organisation Quality of Life Instrument Abbreviated (WHOQOL-BREF) |
| Knight, J, Ayyash, K, Colling, K, et al. (2020) (6) | Investigate relationship between pre-operative frailty and PROMs | Observational cohort study | Not applicable | Edmonton Frail Scale  Clinical Frailty Scale | World Health Organisation Disability Assessment Schedule (WHO DAS 12 item version 2.0) |
| Liu, Z, Han, L, Gahbauer, EA, et al. (2018) (7) | Evaluate joint trajectories of cognition and frailty, and association with PROs | Longitudinal study | Not applicable | Fried Frailty Phenotype | CES-D  Ad-hoc ADL questionnaire  Ad-hoc IADL questionnaire Ad-hoc mobility questionnaire |
| Milne, B, Lucas de Carvalho, J, Ayis, S, et al. (2022) (8) | Evaluate association between preoperative frailty and postoperative disability | Pilot study | Cardiac surgery | Comprehensive Assessment of Frailty | WHO Disability Assessment Schedule WHODAS-12 |
| Tapper, EB, Baki, J, Parikh, ND, et al. (2019) (9) | Investigate associations between frailty, psychoactive medications, cognitive dysfunction and PROs in cirrhosis | Prospective longitudinal | Not applicable | Ad-hoc frailty assessment of physical strength only | SF-8 Pittsburgh Sleep Quality Index (PSQI) Katz Index of Independence in Activity of Daily Living (ADL) |
| Martillo, MA, Dangayach, NS, Tabacof, L, et al. (2021) (10) | Determine characteristics of postintensive care syndrome in cognitive, physical and psychiatric domains of COVID-19 ICU survivors | Descriptive cohort study | Not applicable | Dalhousie Clinical Frailty Scale | EQ-5D-3L Insomnia Severity Index Patient Health Questionnaire-9  PROMIS Short Form v1.0 Fatigue |
| Lieber, SB, Nahid, M, Paget, S, et al. (2022) (11) | Compare two frailty metrics to evaluate differences between frail and non-frail women in people with systemic lupus erythematosus. Investigate whether frailty is associated with self-reported disability | Cross-sectional | Not applicable | Fried Frailty Phenotype FRAIL self-report scale | PROMIS physical function (v2.0)  PROMIS mobility (v2.0)  PROMIS pain behaviour (v1.0)  PROMIS pain interference (v1.1)  PROMIS fatigue (v1.0)  PROMIS anxiety (v1.0)  PROMIS depression (v1.0).  Valued Life Activities VLA  CES-D |
| Tejiram, S, Galet, C, Cartwright, J, et al. (2022) (12) | Evaluate whether global health correlates with worse frailty scores over time, for older adults with acute injury | Prospective observational study | Not applicable | Trauma Specific Frailty Index | Veterans RAND 12 |
| Geense, WW, Zegers, M, Peters, MAA, et al. (2021) (13) | Determine physical, mental and cognitive problems of ICU survivors 1 year after admission | Prospective multicenter cohort study. | Not applicable | Clinical Frailty Scale | Checklist for Individual Strength-Fatigue (8 item version) (CIS-8) Ad-hoc Symptoms Questionnaire  HADS Cognitive Failure Questionnaire (CFQ)  SF-36 |
| Prichard, RA, Zhao, FL, McDonagh, J, et al. (2021) (14) | Compare proxy estimates with patient-reported HRQoL in patients with advanced heart failure | Correlational analysis | Not applicable | Fried Frailty Phenotype (modified for use in heart failure patients) | EQ-5D-5L Depression in Medical Illness DMI-10 |
| Tapper, EB, Kenney, B, Nikirk, S, et al. (2022) (15) | Evaluate Animal Naming Test in sample of older Americans with and without chronic liver disease | Prospective cohort study | Not applicable | Ad-hoc frailty measurements:  ADL IADL Physical assessments | Likert scale for global health |
| Van Bulck, L, Kovacs, AH, Goossens, E, et al. (2022) (16) | Investigate variability and global experience of PROs in adults with congenital heart disease, and give information on epidemiological characteristics and frailty phenotype of older adults with CHD | Cross-sectional global multicentric study | Not applicable | Fried frailty phenotype | RAND-12 Linear Analogue Scale Health Status Patient Health Questionnaire (PHQ-8)  General Anxiety Disorder 7 (GAD-7) Linear Analog Scale Quality of Life  Modified Health Care Climate Questionnaire (HCCQ)  Stigma Scale for Chronic Illness SSCI-8 Illness Identity Questionnaire (IIQ)  Gothenburg Empowerment Scale Multidimensional Perceived Social Support Scale (MSPSS) |
| Jonsson, H, Piscator, E, Israelsson, J, et al. (2022) (17) | Assess how frailty pre-cardiac arrest is associated with long-term survival, neurological function and patient reported outcomes in elderly survivors | Cohort Study | Not applicable | Clinical Frailty Scale (1-7) | EQ-5D-5L Hospital Anxiety and Depression Scale |
| Holeman, TA, Peacock, J, Beckstrom, JL, et al. (2020) (18) | Compare provider assessment of frailty with patient-reported outcome measures of own frailty status, physical function, and social activity level | Cross-sectional observational study | Not applicable | Clinical Frailty Scale  Frail Non-Disabled survey (for patients' use) | PROMIS physical function v1.2  PROMIS social activity level v2.0 PROMIS depression v1.0 |
| Reid, DBC, Daniels, AH, Ailon, T, et al. (2018) (19) | Investigate impact of frailty on postoperative changes in HRQoL | Retrospective analysis of prospective data | Adult Spinal Deformity Surgery | Adult Spinal Deformity Frailty Index | SF-36 (PCS only)  Numeric back pain scores Numeric leg pain scores |
| Kotajarvi, BR, Schafer, MJ, Atkinson, EJ, et al. (2017) (20) | Investigate effect of frailty on PROs before and after surgery | Observational study | Transcatheter (TAVR) or surgical aortic valve replacement (SAVR) | Cardiovascular Health Study Frailty Criteria | Duke Activity Status Index DASI SF-12 Linear analogue self-assessment scale for mental wellbeing  Linear analogue self-assessment scale for physical wellbeing Linear analogue self-assessment scale for emotional wellbeing Linear analogue self-assessment scale for spiritual wellbeing Linear analogue self-assessment scale for level of social activity Linear analogue self-assessment scale for overall quality of life |
| Hanlon, P, Morton, F, Siebert, S, et al. (2022) (21) | Assess prevalence of frailty in rheumatoid arthritis RA and association with disease, all-cause mortality and hospitalisation | Longitudinal cohort study | Not applicable | Cumulative deficit model Fried Frailty phenotype (adapted) | Health Assessment Questionnaire-Disability Index HAQ-DI 0-100 scale from EQ-5D (VAS only) |
| Ponjee, GHM, van de Meerendonk, H, Janssen, MJA, et al. (2021) (22) | Investigate effect of geriatric stewardship on drug-related problems DRPs reported by patients after discharge. | Pre-post study | Geriatric stewardship (inpatient medication reviews) | One or more risk factors for frailty  Participants aged over 65 | Ad-hoc Drug-Related Problems Questionnaire |
| Boumans, R, van Meulen, F, Hindriks, K, et al. (2019) (23) | Investigate effectiveness and acceptability of using Healthcare professional HCP robot assistant. Test hypothesis that a robot can autonomously acquire PROM data from older adults. | Pilot cross-over RCT | Robot administration of PROM questionnaires | TOPICS-Short Form questionnaire (this was used to measure frailty)  Participants aged over 70 | Personal Wellbeing Index PWI  Resilience Scale |
| Zisiopoulou, M, Berkowitsch, A, Seppelt, P, et al. (2021) (24) | Test if novel combination of predictors could improve accuracy of outcome prediction after transfemoral transcatheter aortic valve implantation TAVI. | Prospective study | Transcatheter aortic valve implantation (TAVI) | Clinical Frailty Scale | EQ-5D-5L |
| Boucher, V, Lamontagne, ME, Lee, J, et al. (2019) (25) | Compare acceptability of self-assessment using a tablet in the Emergency Department to a standard assessment by a Research Assistant | Randomised crossover pilot trial | Administration of self-assessment using a tablet | Clinical Frailty Scale | OARS - ADL |
| Zavras, AG, Sullivan, TB, Federico, VP, et al. (2022) (26) | Examine the effect of multifidus muscle quality on patient reported outcomes | Retrospective cohort study | Lateral lumbar interbody fusion (LLIF) | Modified Frailty Index | Visual Analogue Scale (VAS) Back Visual Analogue Scale (VAS) Leg SF12 (PCS) VR12 (PCS) |
| Nicholson, C, Davies, JM, George, R, et al. (2018) (27) | Describe clinical characteristics, symptoms and other concerns of older people with multi-morbidity referred to new community palliative care service. Explore possible implications for service delivery by comparing this population with people receiving standard community-based specialist palliative care. | Cross-sectional study | Palliative care | Older adults (no minimum age) Median age = 88 for BCC group, 78 for control group  Participants were in last year of life and had indicators of poor health such as severe frailty | IPOS |
| McIsaac, DI, Taljaard, M, Bryson, GL, et al. (2016) (28) | Compare predictive accuracy of frailty instruments, consider impact of frailty on PROMs, consider acceptability and feasibility of using frailty instruments in clinical practice | Prospective cohort study | Elective major non-cardiac surgery | Modified Frailty Index  Clinical Frailty Scale | 12-item WHODAS |
| Murata, S, Tsutsui, S, Hashizume, H, et al. (2022) (29) | Determine characteristics to extend healthy life expectancy of older ASD patients | Retrospective analysis | Adult spinal deformity surgery | Adult Spinal Deformity-Frailty Index | SF-36 |
| Quinten, C, Kenis, C, Hamaker, M, et al. (2018) (30) | Assess short and long term effects of chemotherapy on patient reported quality of life and patient vs clinician symptom reporting in older patients with breast cancer | Prospective observational study | Chemotherapy for breast cancer | Geriatric assessment for frailty | Katz ADL Lawton iADL |
| Efficace, F, Boccadoro, M, Palumbo, A, et al. (2018) (31) | Investigate prognostic value of patient self-reported fatigue severity for overall survival in multiple myeloma patients | Prospective observational study | Not applicable | IMWG Frailty Score | Adapted Control Preference Scale  Ad-hoc care satisfaction questionnaire |
| Abdelaziz, HK, Hashmi, I, Taylor, R, et al. (2021) (32) | Investigate impact of transcatheter aortic valve implantation on HRQoL | Prospective cohort study | Transcatheter aortic valve implantation (TAVI) | Rockwood Frailty Score | SF-36 EQ-5D-5L |
| Zisiopoulou, M, Berkowitsch, A, Neuber, R, et al. (2022) (33) | Identify preoperative parameters to predict LoS and PROMs from a scorecard database in patients with significant aortic stenosis who underwent transfemoral aortic valve implantation | Prospective observational cohort study | Transcatheter aortic valve implantation (TAVI) | Clinical Frailty Scale | EQ-5D-5L |
| Orange, ST, Hallsworth, K, Brown, MC, et al. (2022) (34) | Assess feasibility and acceptability of delivering live online home-based group exercise sessions to patients with hepatocellular carcinoma | Non-randomised feasibility study | Home-based virtual exercise intervention | Liver Frailty Index | Functional Assessment of Chronic Illness Therapy-Fatigue (FACIT-F) Activities-specific Balance Confidence scale ABC  HADS  Godin Leisure-Time Exercise Questionnaire - physical activity |
| Bally, ELS, van Grieken, A, Ye, L, et al. (2022) (35) | Assess feasibility and effectiveness of a value-based methodology for integrated care supported by ICT for older people, their informal caregivers, and health and social care practitioners in seven different European settings | Pre-post controlled trial | Implementation of value-based integrated care, supported by ICT | Tilburg Frailty Indicator | For older people:  PROMIS Global-Health v1.2 (PROMIS-10)  UCLA 3-Item Loneliness Scale Modified 10-item Barthel Index Visual Analogue Scale for Fear of Falling SHARE-Frailty (1 item)  International Physical Activity Questionnaire (IPAQ) (1 item)  Medication Risk Questionnaire MRQ-10  EQ-5D-5L  For caregivers or practitioners:  PROMIS Global-Health v1.2 (PROMIS-10) (carer-reported)  iMTA Valuation of Informal Care Questionnaire iVICQ  Zarit Caregiver Burden Assessment (4-item) Adult Social Care Outcomes Toolkit (ASCOT)  Culture of Care Barometer tool  Minnesota Satisfaction Questionnaire - Short Form Copenhagen Burnout Inventory |
| Ekdahl, AW, Wirehn, A-B, Alwin, J, et al. (2015) (36)  Eckerblad, J, Theander, K, Ekdahl, AW, et al. (2016) (37) | Assess costs and effects of CGA in addition to usual care  Follow symptom trajectory of community older people with multimorbidity. Explore effect of symptom burden from ambulatory geriatric care unit based on CGA | RCT | Comprehensive Geriatric Assessment (CGA) | Participants aged over 75 | Barthel index Sense of Security in Care - patients' Evaluation (SEC-P)  EQ-5D-3L Geriatric Depression Scale-15  Memorial Symptom Assessment Scale (MSAS) |
| Fairhall, N, Aggar, C, Kurrle, SE, et al. (2008) (38)  Aggar, C, Ronaldson, S, Cameron, ID. (2012) (39) | Investigate multidisciplinary intervention on frailty   Explore family carers' reactions to caregiving during an intervention targeting community frailty | RCT | Multidisciplinary multifactorial frailty intervention tailored to each participant (e.g. nutritional evaluation, mental health referral, physiotherapy, chronic disease management programs) | Fried frailty phenotype | Barthel Index (100 point version) EQ-5D Geriatric Depression Scale (short form)  Nottingham Extended activities of daily living index Reintegration into Normal Living Index  Exercise Self-Efficacy Scale Goal Attainment Scale Life Space Assessment Ad-hoc social participation item  HADS (carers)  Caregiver Reaction Assessment CRA |
| Mohd Suffian, NI, Adznam, SN, Abu Saad, H, et al. (2020) (40) | Assess the effectiveness of nutritional education and exercise intervention to prevent frailty | Cluster RCT | Nutritional education and exercise intervention | Fried frailty phenotype | Lawton iADL questionnaire (Malay) |
| Jang, IY, Jung, HW, Park, H, et al. (2018) (41) | Evaluate effectiveness of multicomponent intervention on physical function in socioeconomically vulnerable older people | Designed delay study | Multicomponent frailty intervention (exercise, nutrition, depression management,  deprescription, and home hazard reduction) | Fried frailty phenotype | CES-D Ad-hoc ADL Ad-hoc IADL |
| Anderson, BM, Dutton, M, Day, E, et al. (2018) (42) | Measure prevalence and outcomes associated with frailty in a haemodialysis cohort, determine optimum frailty tool, and conduct feasibility study to improve frailty among haemodialysis recipients | RCT | Multidisciplinary frailty intervention (dietetic advice and physiotherapy) | Fried Frailty Phenotype  Clinical Frailty Scale | EQ-5D-3L  PHQ-9 |
| de Nooijer, K, Pivodic, L, Van Den Noortgate, N, et al. (2021) (43)  de Nooijer, K, Van Den Noortgate, N, Pype, P, et al. (2022) (44) | Assess the feasibility and preliminary effectiveness of the Frailty+ intervention  Describe the palliative care symptoms, concerns and wellbeing of older people with frailty and complex care needs upon discharge from hospital to home | RCT | Palliative care intervention | Clinical Frailty Scale (5-7) | IPOS Integrated Palliative Care outcome scale (5 item version)  Ad-hoc symptom item ICECAP supportive care measure Nijmegen Continuity of Care Sense of Security in Care - Patients Palliative Care Outcome Scale IPOS - Views on Care  Sense of security in care - relatives  Family Appraisal of Caregiving Questionnaire for Palliative Care FACQ-PC |
| Ma, L, Zhang, L, Sun, F, et al. (2018) (45) | Explore prevalence of and factors associated with frailty, whether frailty could identify patients at risk of adverse outcomes among older adults with hypertension. | Cross-sectional survey | Not applicable | Rockwood Frailty Index | Ad-hoc ADL Ad-hoc IADL Beijing Longitudinal Study of Ageing physical activity questionnaire BLSA PAQ |
| Bleijenberg, N, Drubbel, I, Ten Dam, VH, et al. (2012) (46) | Evaluate effectiveness of two different frailty interventions | RCT | Screening and monitoring intervention, and nurse-led multidisciplinary intervention program | Groningen Frailty Indicator | Katz ADL index score  RAND-36 and EQ-5D Care-giver burden: Self-Rated Burden (VAS) and Carer-Qol. |
| Chong, E, Zhu, B, Ng SHX, et al. (2022) (47) | Evaluate multicomponent frailty intervention in emergency departments and its effectiveness at improving outcomes | Quasi-experimental study | Multicomponent frailty intervention for emergency department (CGA, frailty education, discharge transition package) | Clinical Frailty Scale (4-6) | Modified Barthel Index MBI Lawton's iADL |
| Yu, R, Tong, C, Leung, G, et al. (2021) (48) | Assess FRAIL scale for identifying frailty in community settings | Correlational analysis | Not applicable | FRAIL scale | SARC-F Lawton iADL scale (Chinese) |
| Liu, LK, Lee, WJ, Chen, LY, et al. (2015) (49) | Evaluate epidemiology of frailty in Taiwan and its association with musculoskeletal health | Longitudinal cohort study | Not applicable | Fried frailty phenotype | Centre for Epidemiologic Studies Depression Scale CES-D |
| Liang, CK, Lee, WJ, Hwang, AC, et al. (2021) (50) | Investigate community intervention for physio-cognitive decline syndrome | Cluster randomised trial | Multi-domain community intervention (physical fitness, cognitive training, nutrition advice) | Cardiovascular Health Study Frailty Criteria (modified) | GDS-5  Lawton's iADL |
| Negm, AM, Kennedy, CC, Ioannidis, G, et al. (2018) (51) | Examine the feasibility of a parallel group RCT comparing preoperative frailty intervention to usual care for older adults undergoing hip or knee replacements | RCT | Multi-modal frailty intervention (exercise, nutrition supplementation, medication reviews) | Fried frailty phenotype | Sarc-F EQ-5D |
| Block, H, Annesley, A, Lockwood, K, et al. (2022) (52) | Determine effectiveness and cost-effectiveness of a frailty intervention on readmissions, frailty and quality of life, compared to usual care | Multisite stepped wedge cluster randomised trial | Multicomponent frailty intervention with two phases: hospital and community | FRAIL scale | EQ-5D-5L |
| Franse, CB, van Grieken, A, Alhambra-Borrás, T, et al. (2018) (53) | Explore effects of coordinated preventive health and social care approach on lifestyle, health and quality of life | Multi-centre pre-post controlled trial | Implementation of Urban Health Centres Europe (UHCE) approach to care, including preventive multidimensional assessment | Tilburg Frailty Indicator SHARE-Frailty instrument | 18-item Groningen activity restriction scale GARS Global Activity Limitation Index GALI SF-12  Full 5 item psychological wellbeing scale of the SF-36 (counted in addition to SF-12)  Falls Efficacy Scale International FES-I  MRQ-10 Jong Gierveld loneliness scale  Ad-hoc physical activity item |
| ClinicalTrials.gov identifier: NCT04715581 (54) | Investigate effect of multimodal prehabilitation on early and long-term outcomes in elderly patients with frailty recovering from cancer surgery | RCT | Multicomponent prehabilitation (nutritional optimisation and exercise training) before surgery for digestive cancer | Clinical Frailty Scale (≥5) | WHOQOL-BREF Pittsburgh Sleep quality index Numeric sleep quality rating scale from 0-10 Numeric pain rating scale from 0-10  International Physical Activity Questionnaire-Long (IPAQ) |
| Serra-Prat, M, Terradellas, M, Lorenzo, I, et al. (2022) (55) | Assess multimodal weight-loss intervention in improving functional performance and reducing frailty risk in obese older adults | RCT | Multimodal weight loss intervention (diet and physical activity) | Fried Frailty phenotype | Barthel index EQ-5D Visual Analogue Scale VAS  Chronic Pain VAS |
| Trialsearch.who.int identifier: ACTRN12612000678897 (56) | Evaluate effect of standardised assessment and management of frailty in elderly respiratory patients on health outcomes compared to usual care | RCT | Assessment for frailty, with Comprehensive Geriatric Assessment referral | Clinical Frailty Scale  Study of Osteoporotic Frailty Scale | Assessment of Quality of Life (AQOL) |
| Kennedy, CC, Novotny, PJ, LeBrasseur, NK, et al. (2019) (57) | Describe risks associated with frailty in patients with chronic obstructive pulmonary disease | Retrospective analysis of RCT data | Not applicable | Fried Frailty phenotype | SF-36 |
| Yousefi, K, Ramdas, KN, Ruiz, JG, et al. (2022) (58) | Evaluate efficacy of Lomecel-B in older adults with frailty | RCT | Lomecel-B drug intervention – allogeneic  medicinal signaling cell (MSC) formulation | Clinical Frailty Scale (5-6) Cardiovascular Health Study Frailty Criteria | PROMIS Physical Function - Short Form 20a PROMIS Physical Function - mobility PROMIS Physical Function - upper extremity Falls Efficacy Scale-International Geriatric Depression Scale-Short Form Sexual Quality of Life-Female (SQOL-F)  International Index of Erectile Function (IIEF) |
| Trialsearch.who.int identifier: ISRCTN16588124 (59) | Collect observational data on ageing and a recruitment platform for research with older people | Observational cohort study | Not applicable | electronic Frailty Index | Barthel index  Nottingham Extended Activities of Daily Living EQ-5D  SF-36  Geriatric Pain Measure Geriatric Depression Scale De Jong Gierveld Loneliness Scale |
| ClinicalTrials.gov identifier: NCT02305433 (60) | Study the effects of intensive home-based physiotherapy in people with an operated hip fracture or with signs of frailty | RCT | Home-based physiotherapy | Fried Frailty phenotype | 15D  Falls Efficacy Scale - International (FES-I) Social Provision Scale (SPS) GDS-15 |
| Gilmore, N, Kehoe, L, Bauer, J, et al. (2021) (61) | Examine relationship between frailty and emotional health in older patients with cancer | Secondary analysis of cluster RCT data | Not applicable | Deficit Accumulation Index | Ad-hoc ADL Questionnaire Ad-hoc IADL Questionnaire OARS - Physical Health Comorbidity Questionnaire (15 items used)  GDS Depression GAD-7 Generalised Anxiety Disorder-7 Ad-hoc Social Activities Questionnaire MOS Social Support Survey (4 items used) |
| McIsaac, DI, Fergusson, DA, Khadaroo, R, et al. (2022) (62) | Compare home-based prehabilitation vs standard care among patients with frailty having surgery | Multicentre Randomised Trial | Home-based prehabilitation for elective major surgery | Clinical Frailty Scale | WHODAS  Katz index  EQ-5D-5L |
| Trialsearch.who.int identifier: ACTRN12620001173987 (63) | Evaluate effectiveness of nurse-led volunteer support interventions and technology driven pain assessment compared with standard care | Cluster RCT | Nurse-led volunteer support interventions and technology-based pain assessment | Modified-Reported Edmonton Frail Scale | Numerical pain rating Australian Quality of Life questionnaire AQoL |
| Hirai, K, Homma, T, Matsunaga, T, et al. (2020) (64) | Examine efficacy of Ninjin'yoeito medicine in frailty or prefrailty patients with chronic obstructive pulmonary disease | RCT | Ninjin'yoeito (traditional Japanese medicine) drug intervention | Kihon Checklist | HADS |
| Trialsearch.who.int identifier: IRCT20201108049310N1 (65) | Determine the impact of lifestyle education based on pender model on frailty outcomes in community-dwelling older adults | RCT | Healthy lifestyle education | Tilberg Frailty Scale | Katz ADL Falls Self-Efficacy Scale Questionnaire International physical activity questionnaire IPAQ |
| Villareal, DT, Banks, M, Sinacore, DR, et al. (2006) (66) | Evaluate the effects of weight loss and exercise therapy on physical function and body composition in obese older adults | RCT | Diet and exercise therapy for weight loss | Participants aged over 65  Frailty assessment:  Physical Performance Test score  Oxygen consumption Self-reported ADL and IADL in Functional Status Questionnaire | SF-36 |
| Young, H, March, D, Highton, P, et al. (2021) (67) | Determine whether a randomised controlled trial of intradialytic exercise is feasible for frail haemodialysis patients | Mixed-methods randomised controlled feasibility study | Exercise intervention for haemodialysis patients | Clinical Frailty Scale (4-7) | DASI SF-12 HADS Exercise Self-Efficacy Scale ESES |
| Chan, DC, Tsou, HH, Yang, RS, et al. (2012) (68) | To determine the effects of two interventions on frailty and other outcomes, and explore the feasibility of a larger trial | RCT | Exercise and nutritional program, and problem-solving therapy | Chinese Canadian Study CFS Clinical Frailty Scale | Barthel Index  PRIME-MD Primary Care Evaluation for Mental Disorders  EQ-5D |
| Griffin, AC, O'Neill, A, O'Connor, M, et al. (2020) (69) | Determine the malnutrition prevalence, associated factors on presentation and post-discharge adverse outcomes among older adults in emergency departments | Secondary analysis of RCT data | Not applicable | Clinical Frailty Scale | EQ-5D-5L Barthel Index |
| Gomes, GCV, Simões, MDS, Lin, SM, et al. (2018) (70) | Evaluate the feasibility, safety and acceptability of playing Nintendo Wii Fit PlusTM interactive video games in frail and pre-frail older adults | Randomised controlled parallel-group feasibility trial | Nintendo Wii Fit Plus™ interactive video games | Fried Frailty Phenotype | GDS-15 Depression FES-I fear of falling |
| Tompkins, BA, DiFede, DL, Khan, A, et al. (2017) (71) | Investigate whether the use of exogenous human allogeneic mesenchymal stem cells can reverse signs and symptoms of frailty in older adults | RCT | Human allogeneic mesenchymal stem cells (allo-hMSCs) delivered intravenously | Canadian Clinical Frailty Scale (4-7) | Sexual Quality of Life-Female SQOL-F  International Index of Erectile Function IIEF  CHAMPS questionnaire Exhaustion-Multidimensional Fatigue Inventory |
| Trialsearch.who.int identifier: ACTRN12620000271909 (72) | Determine the impact of frailty in older people with haematological malignancies | RCT | Geriatric assessment | Participants aged over 65 | Ad-hoc ADL Ad-hoc IADL |
| Applegate, WB, Miller, ST, Graney, MJ, et al. (1990) (73) | Determine effect of treatment in a geriatric assessment unit on physical function, institutionalisation rate and mortality of elderly patients | RCT | Admission to geriatric assessment unit in community rehabilitation hospital | Participants aged over 65 | Ad-hoc ADL CES-D |
| Hogan, DB, Fox, RA (1990) (74) | Assess usefulness of geriatric consultation teams in acute-care settings | Prospective controlled trial | Geriatric Consultation Team | Participants aged over 75 | Barthel Index |
| Winograd, CH, Gerety, MB, Lai, NA (1993) (75) | Determine effectiveness of inpatient interdisciplinary geriatric consultation during hospitalisation for frail elderly participants | RCT | Inpatient Geriatric Consultation | Over 65 One of five criteria for frailty, including ADL dependence, confusion or disabling chronic illnesses | Physical Self-Maintenance Scale and Lawton's Instrumental Activities of Daily Living (counted individually) Philadelphia Geriatric Center Morale Scale |
| Karppi, P (1995) (76) | Test value of bringing community-dwelling patients into hospital geriatric assessment unit | RCT | Admission to geriatric inpatient unit for assessment and rehabilitation | Participants aged over 65 | Katz ADL index Lawton and Brody Iadl Scale Zung Self-Rating Depression Scale SDS  Health Likert Scale |
| Cohen, HJ, Feussner, JR, Weinberger, M, et al. (2002) (77) | Evaluate effect of inpatient units and outpatient clinics on survival and functional stats | RCT | Inpatient geriatric unit, and outpatient geriatric unit | Participants aged over 65 Two or more criteria for frailty, including ADL dependence, history of falls, malnutrition and dementia | SF-36 Katz ADL Fillenbaum iADL - OARS |
| Saltvedt, I, Jordhøy, M, Opdahl, Mo ES, et al. (2006) (78) | Evaluate effect of treatment in a geriatric evaluation and management unit on wellbeing | RCT | Geriatric Evaluation and Management Unit (GEMU) | Participants aged over 75 At least one of Winograd's frailty screening criteria | Barthel Index Lawton Instrumental Activity of Daily Living instrument Philadelphia Geriatric Centre Morale Scale |
| Hinkka, K, Karppi, SL, Pohjolainen, T, et al. (2007) (79)  Ollonqvist, K, Aaltonen, T, Karppi, S-L, et al. (2008) (80)  Kehusmaa, S, Autti-Rämö, I, Valaste, M, et al. (2010) (81) | Study feasibility and one-year effects on subjective health and symptoms of a network-based geriatric rehabilitation intervention for frail elderly people  Investigate effects on formal support  Evaluate cost-effectiveness of support | RCT | Multidisciplinary geriatric rehabilitation programme (comprehensive geriatric assessment, home assessment, group-based activities such as physical activity) | Participants aged over 65 Progressively decreasing functional ability Risk of institutionalisation within 2 years | Health Likert Scale Pain Visual Analogue Scale Ad-hoc Symptoms Questionnaire GDS Ad-hoc ADL Ad-hoc IADL 15D |
| Kircher, TTJ, Wormstall, H, Müller, PH, et al. (2007) (82) | Examine effects of inpatient geriatric consultation service | RCT | Comprehensive geriatric assessment and management | Participants aged over 65 At least two criteria indicating functional disability | Philadelphia Geriatric Centre Morale Scale PGCMS  Barthel Index  Geriatric Depression Scale |
| Torres-Sánchez, I, Valenza, MC, Cabrera-Martos, I, et al. (2017) (83) | Determine whether pedal exerciser intervention can reduce disability in frail older patients with chronic obstructive pulmonary disease during hospitalisation | RCT | Exercise intervention | Brief Frailty Index (3-5) | Barthel Index Modified Baecke physical activity questionnaire |
| Bernabei, R, Landi, F, Gambassi, G, et al. (1998) (84) | Evaluate impact of programme of integrated social and medical care among frail elderly people living in the community | RCT | Case management and  care planning by community geriatric evaluation  unit and general practitioners | Participants aged over 65 | Geriatric Depression Scale |
| Shannon, GR, Wilber, KH, Allen, D (2006) (85) | Determine whether telephone care management for high-risk Medicare health maintenance organisation health plan enrolees can reduce medical utilization | RCT | Telephone care-management intervention | Participants aged over 65 Met algorithm criteria for high healthcare usage | Katz index of independence  Lawton's iADL |
| Markle-Reid, M, Weir, R, Browne, G, et al. (2006) (86) | Evaluate comparative effects and costs of proactive nursing health promotion intervention in addition to usual home care for older people compared with usual home care services alone | RCT | Proactive nursing health promotion intervention (health assessment, education about management of illness, coordination of community services, use of empowerment strategies) | Participants aged over 75 Required assistance with personal care | SF-36 CES-D Center for Epidemiological Studies in Depression Scale Personal Resource Questionnaire 85 (part two) |
| Béland, F, Bergman, H, Lebel, P, et al. (2006) (87) | Compare SIPA with usual care for vulnerable community-dwelling elderly persons | RCT | Implementation of System of Integrated Care for Older Persons (SIPA): multidisciplinary teams providing integrated care and interventions in community | Participants aged over 64 SMAF disability score of “moderate” or above | GDS Nagi Scale Barthel Index OARS – ADL (IADL only)  Client Satisfaction Questionnaire (CSQ-8)   Caregivers:  CSQ-8 Zarit Scale |
| Gagnon, AJ, Schein, C, McVey, L, et al. (1999) (88) | Compare effects of nurse case management with usual care provided to community-dwelling frail older people | RCT | Nurse case management: coordination and provision of healthcare services by nurses in and out of hospital | Participants aged over 70 Require assistance with at least one ADL or two IADL activities At risk of hospital admission | SF-36 CSQ-8 OARS - ADL |
| Rockwood, K, Stadnyk, K, Carver, D, et al. (2000) (89) | Test CGA as an adjunct to usual care | RCT | Comprehensive Geriatric Assessment | Participants with at least one condition indicating frailty (including frequent physician contact, polypharmacy, multiple medical problems) | Barthel Index Physical Self-Maintenance Scale (PSMS) and Lawton-Brody IADL scale (counted individually) Goal Attainment Scale (GAS) |
| Newcomer, R, Maravilla, V, Faculjak, P, et al. (2004) (90) | Measure patient outcomes 12 months after preventive case management was implemented | RCT | Prevention-oriented case management program by nurses | Participants aged over 80, or aged over 65 with at least one qualifying condition | SF-12 Katz ADL  Lawton and Brody iADL |
| Leveille, SG, Wagner, EH, Davis, C, et al. (1998) (91) | Evaluate impact of one-year senior center-based chronic illness self-management and disability prevention program | RCT | Multi-component disability prevention and disease self-management program, targeted to individuals | Participants aged over 70 with at least one chronic condition | SF-36 HAQ Health Assessment Questionnaire  CES-D Depression Scale PASE Physical Activity Scale for the Elderly |
| Ambrosius, WT, Sink, KM, Foy, CG, et al. (2014) (92) | Describe the design of the SPRINT study which compared two strategies for treating systolic blood pressure | Multicentre RCT | Standard and intensive programs of hypertension treatment by antihypertensive agents | Participants aged over 50, with subgroup over 75 | VR-12 EQ-5D-3L PHQ-9 FES-I Female Sexual Function Index FSFI International Index of Erectile Function 5-item version IIEF-5 |
| Trialsearch.who.int identifier: ACTRN12620000607976 (93) | Investigate whether computerised cognitive training improves the frailty status of older hospitalised patients | RCT | Computerised cognitive training | Edmonton Frail Scale | EQ-5D |

# Appendix 3 – Complete List of Included PROMs

| **Measure name** | **Frequency of usage** | **Citation** | **Domain** | **Details** |
| --- | --- | --- | --- | --- |
| Barthel Index | 14 | Mahoney, FI, Barthel, DW (1965) (94) | Activities of Daily Living | 10 items assessing activities of daily living (ADL), with 3 levels each |
| Lawton-Brody Instrumental Activities of Daily Living (IADL) scale) | 11 | Lawton, MP, Brody, EM (1969) (95) | Activities of Daily Living | Assesses 8 activities including using the telephone, housekeeping and transportation Scoring is different for males and females  Same paper as the Physical Self-Maintenance Scale (PSMS) |
| Katz Index of Activities of Daily Living (ADL) | 9 | Katz, S, Downs, TD, Cash HR, et al. (1970) (96) | Activities of Daily Living | Assesses 6 activities of daily living on a 2-point scale, including bathing, feeding and toileting |
| Ad-hoc Activities of Daily Living (ADL) Measures | 7 | Ollonqvist, K, Aaltonen, T, Karppi, S-L, et al. (2008) (80) Gilmore, N, Kehoe, L, Bauer, J, et al. (2021) (61)  Trialsearch.who.int identifier: ACTRN12620000271909 (72) Applegate, WB, Miller, ST, Graney, MJ, et al. (1990) (73) Liu, Z, Han, L, Gahbauer, EA, et al. (2018) (7)  Jang, IY, Jung, HW, Park, H, et al. (2018) (41)  Ma, L, Zhang, L, Sun, F, et al. (2018) (45) | Activities of Daily Living | Variable between studies Measures of ADL that are not referenced to particular instruments |
| Ad-hoc Instrumental Activities of Daily Living (IADL) Measures | 6 | Ollonqvist, K, Aaltonen, T, Karppi, S-L, et al. (2008) (80) Gilmore, N, Kehoe, L, Bauer, J, et al. (2021) (61) Trialsearch.who.int identifier: ACTRN12620000271909 (72)  Liu, Z, Han, L, Gahbauer, EA, et al. (2018) (7) Jang, IY, Jung, HW, Park, H, et al. (2018) (41) Ma, L, Zhang, L, Sun, F, et al. (2018) (45) | Activities of Daily Living | Variable between studies Measures of IADL that are not referenced to particular instruments |
| PROMIS Physical Function (including Upper Extremity and Mobility) | 6 | HealthMeasures (2022) (97) | Activities of Daily Living | Items assess individual's ability to perform physical tasks in daily life, on a 5-point scale  Multiple versions exist, including Physical Function, Physical Function - Upper Extremity, and Physical Function - Mobility |
| Older Americans Resources and Services (OARS) - Activities of Daily Living | 4 | Fillenbaum (2005) (98) | Activities of Daily Living | Subsection of the OARS Multidimensional Functional Assessment Questionnaire (MFAQ)  Contains 7 items on Instrumental Activities of Daily Living (IADL) and 8 items on Physical Activities of Daily Living (ADL)  5-item subscale of IADL also exists |
| WHODAS | 4 | World Health Organisation (2012) (99) | Activities of Daily Living | Assesses 6 domains, including mobility, self-care and social interaction  36-item and 12-item versions exist (WHODAS-36 and WHODAS-12) |
| Duke Activity Status Index (DASI) | 2 | Hlatky, MA, Boineau, RE, Higginbotham, MB, et al. (1989) (100) | Activities of Daily Living | 12 items assessing activities More physically demanding activities are weighted with higher scores |
| Groningen Activity Restriction Scale (GARS) | 1 | Suurmeijer, TP, Doeglas, DM, Moum, T, et al. (1994) (101) | Activities of Daily Living | 18 items on a 4-point scale  11 items assess Activities of Daily Living (ADL) and 7 items assess Instrumental Activities of Daily Living (IADL) |
| Health Assessment Questionnaire-Disability Index (HAQ-DI) | 2 | Hawley, DJ, Wolfe, F (1992) (102) | Activities of Daily Living | 8 categories of activities, including dressing and grooming, reach, and activities  Assesses 20 specific activities and support needed  Also contains scales from 0-100 measuring pain and health |
| Nottingham Extended Activities of Daily Living | 2 | Nouri, F, Lincoln, N (1987) (103) | Activities of Daily Living | 22 items on a 4-point scale assessing instrumental activities of daily living (IADL), including crossing roads, making a hot drink and writing letters |
| SARC-F | 2 | Malmstrom, TK, Morley, JE (2013) (104) | Activities of Daily Living | 5 items measuring presence and severity of sarcopenia symptoms, with 3 levels each |
| Physical Self-Maintenance Scale (PSMS) | 2 | Lawton, MP, Brody, EM (1969) (95) | Activities of Daily Living | Assesses 6 activities with scores of 1 or 0, including toilet, feeding and dressing  Same paper as the Lawton-Brody Instrumental Activities of Daily Living (IADL) scale |
| Ad-hoc mobility questionnaire | 1 | Liu, Z, Han, L, Gahbauer, EA, et al. (2018) (7) | Activities of Daily Living | Participants asked about assistance for three items: walking, climbing stairs, and carrying 10lb Also asked if they had driven a car in the last month |
| Nagi Scale of Disability | 1 | Nagi, SZ (1976) (105) | Activities of Daily Living | 15 items on a 4-point scale  Two factors: Physical Performance Scale and Emotional Performance Scale |
| Valued Life Activities (VLA) | 1 | Katz, P, Morris, A, Trupin, L, et al. (2008) (106) | Activities of Daily Living | 21 items on a 4-point scale assessing disability for activities that are important in life 3 categories: obligatory, committed, and discretionary activities |
| Life Space Assessment | 1 | Peel, C, Sawyer Baker, P, Roth, DL, et al. (2005) (107) | Activities of Daily Living | 5 items assessing mobility inside and outside the house, and frequency and independence |
| Adult Social Care Outcomes Toolkit (ASCOT) for carers | 1 | Netten, A, Burge, P, Malley, J, et al. (2012) (108) | Carer: Autonomy and Control | 7 items with 4 levels  ASCOT self-completion tools exist for both patients and carers |
| Zarit Caregiver Burden Assessment | 2 | Zarit, SH, Reever, KE, Bach-Peterson, J (1980) (109) | Carer: Caregiver Burden | 22 item, 12 item and 4 item versions exist |
| iMTA Valuation of Informal Care Questionnaire (iVICQ) | 1 | Bouwmans, C, Krol, M, Severens, H, et al. (2015) (110) | Carer: Caregiver Burden | Reports carers' time spent on caregiving activities, to provide estimates for societal costs Contains 7 sections, including the CarerQol |
| Self-Rated Burden Scale (Visual Analogue) | 1 | Bleijenberg, N, Drubbel, I, Ten Dam, VH, et al. (2012) (46) | Carer: Caregiver burden | Caregivers asked to rate perceived burden due to caregiving |
| Caregiver Reaction Assessment (CRA) | 1 | Petrinec, A, Burant, C, Douglas, S (2016) (111) | Carer: Caregiver burden | 24 items covering 5 dimensions on caregiving. Each item is on a 5-point scale |
| Carer-Qol | 1 | Brouwer, WB, van Exel, NJ, van Gorp, B, et al. (2006) (112) | Carer: Caregiver burden | Subsection of the iVICQ  7 items describing caregiving situations, each with 3 levels Also includes Visual Analogue Scale (0-10) |
| Family Appraisal of Caregiving Questionnaire for Palliative Care (FACQ-PC) | 1 | Cooper, B, Kinsella, GJ, Picton, C. (2006) (113) | Carer: Caregiver burden | 26 items on a 5-point scale 4 subscales: caregiver strain, positive caregiving appraisals, caregiver distress, and family wellbeing |
| Client Satisfaction Questionnaire (CSQ-8) (carer-reported) (counted as the same measure as for patients) | 1 | Larsen, DL, Attkisson, CC, Hargreaves, WA, et al. (1979) (114) | Carer: Healthcare Satisfaction | Caregivers of older adults assessed how satisfied they were with formal healthcare received in an intervention |
| Sense of Security in Care – Relatives | 1 | Krevers, B, Milberg, A (2015) (115) | Carer: Healthcare Satisfaction | 17 items on a 6-point scale assessing patient's healthcare from relative's perspective 3 components: care interaction, mastery, and patient situation |
| Hospital Anxiety and Depression Scale (HADS) (carer-reported) (counted as the same measure as for patients) | 1 | Zigmond, AS, Snaith, RP (1983) (116) | Carer: Mood and Emotional Health | 7 items on anxiety and 7 items on depression on a 4-point scale Used to assess carer's health |
| PROMIS Global Health (v1.2) (carer-reported) (counted as the same measure as for patients) | 1 | HealthMeasures (2022) (97) | Carer: Physical Health | 9 items on a 5-point scale assessing global health, and a Visual Analogue Scale from 0-10 Was used to report carer health as well as patient health |
| Copenhagen Burnout Inventory (carer) | 1 | Kristensen, TS, Borritz, M, Villadsen, E, et al. (2005) (117) | Carer: Work Satisfaction | 19 items Assesses burnout for carers across three domains: personal, work-related and client-related burnout |
| Minnesota Satisfaction Questionnaire - Short Form (carer) | 1 | Weiss, DJ, Dawis, RV, England, GW. (1967) (118) | Carer: Work Satisfaction | 20 items on a 5-point scale, measuring healthcare workers' satisfaction with their jobs |
| Culture of Care Barometer tool | 1 | Rafferty, AM, Philippou, J, Fitzpatrick, JM, et al. (2017) (119) | Carer: Work Satisfaction | 30 items with 5 levels each that assess workplace culture |
| PROMIS Fatigue (v1.0) | 2 | HealthMeasures (2022) (97) | Fatigue | 95 items on a 5-point scale assessing fatigue symptoms ranging in severity |
| Checklist Individual Strength (CIS) | 1 | Vercoulen, JH, Swanink, CM, Fennis, JF, et al. (1994) (120) | Fatigue | Assesses fatigue on a 7-point scale 20-item and 8-item versions exist |
| Fatigue Scale (Visual Analogue) | 1 | Justice, JN, Nambiar, AM, Tchkonia, T, et al. (2019) (2) | Fatigue | Assesses fatigue on a visual analogue scale |
| Multidimensional Fatigue Inventory (MFI) | 1 | Smets, EM, Garssen, B, Bonke, B, et al. (1995) (121) | Fatigue | 20 items with 5 levels each, assessing fatigue |
| Fatigue Severity Scale (FSS) | 1 | Valko, PO, Bassetti, CL, Bloch, KE, et al. (2008) (122) | Fatigue | 9 items on a 7-point scale Also includes a Visual Analogue Scale from 0-10 |
| Functional Assessment of Chronic Illness Therapy-Fatigue (FACIT-F) | 1 | Webster, K, Cella, D, Yost, K (2003) (123) | Fatigue | 40 items on a 5 point scale assessing 5 categories: physical wellbeing, social/family wellbeing, emotional wellbeing, functional wellbeing, and additional concerns Distinct from FACIT Fatigue Scale, which has 13 items |
| Pittsburgh Fatigability Scale (PFS) | 1 | Glynn, NW, Santanasto, AJ, Simonsick, EM, et al. (2015) (124) | Fatigue | Assesses 4 components: sedentary activity, lifestyle or light-intensity activity, moderate- to high-intensity activity, and social activity   26 item and 10 item versions exist |
| Client Satisfaction Questionnaire (CSQ-8) | 2 | Larsen, DL, Attkisson, CC, Hargreaves, WA, et al. (1979) (114) | Healthcare Satisfaction | 8 items, with 4 levels each Assesses patient or client satisfaction with the care received |
| Sense of Security in Care - Patients | 2 | Krevers, B, Milberg, A (2014) (125) | Healthcare Satisfaction | 15 items on a 6-point scale assessing patients' feelings of security about healthcare  3 components: care interaction, identity, and mastery |
| Ad-hoc patient satisfaction questionnaire | 1 | Efficace, F, Boccadoro, M, Palumbo, A, et al. (2018) (31) | Healthcare Satisfaction | Patients rate satisfaction with care and with overall decision-making process |
| Short Form Health Survey (SF-36, SF-12 and SF-8)  (Includes full and partial usage) | 21 | Ware, JE, Jr., Sherbourne, CD (1992) (126) | Health-Related Quality of Life | Assesses 8 domains, including physical health limitations, mental health, and vitality  36-item, 12-item and 8-item versions exist (SF-36, SF-12 and SF-8)  Contains the same items as Veterans RAND Health Survey but scored differently |
| EQ-5D  (Including full and partial usage) | 21 | Rabin, R, de Charro, F (2001) (127) | Health-Related Quality of Life | 5 items. 3-Level and 5-Level versions exist (EQ-5D-3L and EQ-5D-5L)  Also contains Visual Analogue Scale from 0-100 |
| Health Scale | 5 | Hinkka, K, Karppi, SL, Pohjolainen, T, et al. (2007) (79)  Tapper, EB, Kenney, B, Nikirk, S, et al. (2022) (15)  Karppi, P (1995) (76)  Van Bulck, L, Kovacs, AH, Goossens, E, et al. (2022) (16) Kotajarvi, BR, Schafer, MJ, Atkinson, EJ, et al. (2017) (20) | Health-Related Quality of Life | Variable between studies – 2 Linear Analogue Scales and 3 Likert Scales Participants asked to rate their health or physical wellbeing on a scale |
| Veterans Rand Health Survey (VR-36 and VR-12) | 5 | Hays, RD, Sherbourne, CD, Mazel, RM. (1993) (128) | Health-Related Quality of Life | Assesses 8 domains, including physical health limitations, mental health, and vitality  36-item and 12-item versions exist (VR-36 and VR-12)  Contains the same items as Short Form Health Survey but scored differently |
| Quality of Life Scale | 4 | Van Bulck, L, Kovacs, AH, Goossens, E, et al. (2022) (16)  Kotajarvi, BR, Schafer, MJ, Atkinson, EJ, et al. (2017) (20)  Justice, JN, Nambiar, AM, Tchkonia, T, et al. (2019) (2) | Health-Related Quality of Life | Participants asked to rate overall quality of life or change in overall quality of life  Linear analogue scales, visual analogue scales and Likert scales used |
| Ad-hoc Symptoms Questionnaire | 3 | Hinkka, K, Karppi, SL, Pohjolainen, T, et al. (2007) (79) Geense, WW, Zegers, M, Peters, MAA, et al. (2021) (13) de Nooijer, K, Pivodic, L, Van Den Noortgate, N, et al. (2021) (43) | Health-Related Quality of Life | Variable between studies Participants asked to assess physical symptoms |
| 15D | 2 | Sintonen, H (2001) (129) | Health-Related Quality of Life | 15 items with 5 levels each Includes items on breathing, sleeping, mobility and mental health |
| Assessment of Quality of Life (AQOL) (sometimes also called Australian Quality of Life Assessment) | 2 | Hawthorne, G, Richardson, J, Osborne, R (1999) (130) | Health-Related Quality of Life | 15 items with 4 levels each Measures 5 domains, including independent living and social relationships |
| WHOQOL-BREF | 2 | World Health Organization (‎2004) (131) | Health-Related Quality of Life | 26 items on a 5-point scale, measuring quality of life across multiple areas of life 100-item version (WHOQOL-100) also exists |
| Memorial Symptom Assessment Scale | 1 | Portenoy, R K, Thaler, H T, Kornblith, A B, et al. (1994) (132) | Health-Related Quality of Life | Assesses the presence and severity of physical and mental symptoms, including dry mouth, nausea and worrying, and the distress caused by each symptom  Section 1 has 24 items and Section 2 has 8 items |
| Global Activity Limitation Index (GALI) | 1 | van Oyen, H, Van der Heyden, J, Perenboom, R, et al. (2006) (133) | Health-Related Quality of Life | 1 item on a 3-point scale assessing activity limitation due to a health problem |
| Older Americans Resources and Services (OARS) - Comorbidity Questionnaire | 1 | Fillenbaum (2005) (98) | Health-Related Quality of Life | Subsection of the OARS Multidimensional Functional Assessment Questionnaire (MFAQ)  Asks respondents about the presence of 26 illnesses, and their perceived effect on activities on a 3-point scale |
| PROMIS Global Health (v1.2) | 1 | HealthMeasures (2022) (97) | Health-Related Quality of Life | 9 items on a 5-point scale assessing global health, and a Visual Analogue Scale from 0-10 |
| Hyland Scale | 1 | Hyland, ME, Sodergren, SC (1996) (134) | Health-Related Quality of Life | Visual Analogue Scale on a 101 point scale  0 = “perfect quality of life”  100 = “might as well be dead” |
| Personal Wellbeing Index (PWI) | 1 | International Wellbeing Group (2013) (135) | Health-Related Quality of Life | 9 items (including 2 optional items) assessing overall quality of life, including health, relationships and goals |
| De Jong Gierveld Loneliness Scale | 2 | De Jong Gierveld, J, Van Tilburg, T (2006) (136) | Loneliness and Isolation | Assesses two domains: social loneliness and emotional loneliness 11 item and 6 item versions exist |
| UCLA 3-Item Loneliness Scale | 1 | Hughes, ME, Waite, LJ, Hawkley, LC, et al. (2004) (137) | Loneliness and Isolation | 3 items on a 3-point scale measuring loneliness  20 item version also exists |
| MRQ-10 | 2 | Barenholtz Levy, H (2003) (138) | Medication Satisfaction/Burden | 10 items  Assesses risk factors for inappropriate medication usage, including number of medications and practical problems |
| Ad-hoc Drug-Related Problems Questionnaire | 1 | Ponjee, GHM, van de Meerendonk, H, Janssen, MJA, et al. (2021) (22) | Medication Satisfaction/Burden | 4 sections of the questionnaire, addressing presence of symptoms, practical problems with taking medication, questions about medication and satisfaction with medication  Questionnaire is adapted from the validated questionnaire by Willeboordse, F, Grundeken, LH, van den Eijkel, LP, et al. (2016) (139) but is not identical to this. It therefore appears to be unvalidated |
| Falls Efficacy Scale International (FES-I) | 6 | Yardley, L, Beyer, N, Hauer, K, et al. (2005) (140) | Mobility Confidence | 16 items on a 4-point scale Assesses fear of falling while performing specific activities |
| Exercise Self-Efficacy Scale (ESES) | 2 | Kroll, T, Kehn, M, Ho, PS, et al. (2007) (141) | Mobility Confidence | 10 items with 4 levels each, assessing confidence at performing physical activities |
| Activities-Specific Balance Confidence scale (ABC) | 1 | Powell, LE, Myers, AM (1995) (142) | Mobility Confidence | 16 items, each on an 11-point scale  Contextualises mobility with specific activities |
| Fear of Falling Scale (Visual Analogue) | 1 | Bally, ELS, van Grieken, A, Ye, L, et al. (2022) (35) | Mobility Confidence | Participants asked to rate fear of falling |
| Geriatric Depression Scale (GDS) | 12 | Yesavage, JA, Brink, TL, Rose, TL, et al. (1982) (143) | Mood and Emotional Health | Assesses symptoms of depression on a 2-point scale  30-item, 15-item and 5-item versions exist |
| Center for Epidemiologic Studies Depression (CES-D) Scale | 7 | Radloff, LS (1977) (144) | Mood and Emotional Health | 20 items assessing symptoms of depression, with 4 levels each |
| Hospital Anxiety and Depression Scale (HADS) | 6 | Zigmond, AS, Snaith, RP (1983) (116) | Mood and Emotional Health | 7 items on anxiety and 7 items on depression on a 4-point scale |
| Patient Health Questionnaire (PHQ) | 4 | Kroenke, K, Spitzer, RL, Williams, JB (2001) (145) | Mood and Emotional Health | Assess depression on a 4-point scale 9-item and 8-item versions exist (PHQ-9 and PHQ-8) |
| Mental/Emotional Wellbeing Scale (Linear Analogue) | 2 | Kotajarvi, BR, Schafer, MJ, Atkinson, EJ, et al. (2017) (20) | Mood and Emotional Health | Participants asked to rate mental or emotional wellbeing |
| Generalised Anxiety Disorder 7-item (GAD-7) | 2 | Spitzer, RL, Kroenke, K, Williams, JBW, et al. (2006) (146) | Mood and Emotional Health | 7 items on a 4-point scale assessing the presence of symptoms of anxiety |
| PROMIS Emotional Distress - Depression (v1.0) | 2 | HealthMeasures (2022) (97) | Mood and Emotional Health | 28 items on a 5-point scale on symptoms of depression |
| Depression in Medical Illness (DMI) | 1 | Parker, G, Hilton, T, Bains, J, et al. (2002) (147) | Mood and Emotional Health | Assess depression symptoms in those with a physical illness on a 4-point scale 18-item and 10-item versions exist (DMI-18 and DMI-10) |
| Primary Care Evaluation for Mental Disorders (PRIME-MD) | 1 | Spitzer, RL, Williams, JB, Kroenke, K, et al. (1994) (148) | Mood and Emotional Health | 26 items, as well as a scale for overall health  Evaluates mood, anxiety, somatoform and alcohol consumption |
| PROMIS Emotional Distress - Anxiety (v1.0) | 1 | HealthMeasures (2022) (97) | Mood and Emotional Health | 29 items on a 5-point scale on symptoms of anxiety |
| Zung Self-Rating Depression Scale SDS | 1 | Zung, WWK (1965) (149) | Mood and Emotional Health | 20 items assessing depression, with 4 levels each |
| Philadelphia Geriatric Centre Morale Scale | 3 | Lawton, MP (1975) (150) | Other - Morale | 17 items on a 2-point scale  3 factors emerge from the scale: agitation, attitude toward own aging, and lonely dissatisfaction |
| Resilience Scale | 1 | Wagnild, GM, Young, HM (1993) (151) | Other - Perceived Resilience | Assesses resilience to stressful situations  25 items each on a 7-point scale |
| Stigma Scale for Chronic Illness - 8 item version (SSCI-8) | 1 | Molina, Y, Choi, SW, Cella, D, et al. (2013) (152) | Other - Perceived Stigma | 8 items on a 5-point scale assessing perceived stigma about their illness 24 item version also exists |
| Goal Attainment Scale (GAS) | 2 | Kiresuk, TJ, Smith, A, Cardillo, JE (eds.). (1994) (153) | Other - Personal Goals | Patient sets individualised goals over a set time period, weighted by importance and with expected outcomes stated. At follow-up, patient completion of goals is assessed and scored |
| Illness Identity Questionnaire (IIQ) | 1 | Oris, L, Luyckx, K, Rassart, J, et al. (2018) (154) | Other - Self-Identity | 25 items on a 5-point scale  Assesses 4 identity states: engulfment, rejection, acceptance and enrichment |
| Spiritual Wellbeing Scale (Linear Analogue) | 1 | Kotajarvi, BR, Schafer, MJ, Atkinson, EJ, et al. (2017) (20) | Other - Spiritual Wellbeing | Participants asked to rate spiritual wellbeing |
| Pain Scale | 8 | Reid, DBC, Daniels, AH, Ailon, T, et al. (2018) (19)  Zavras, AG, Sullivan, TB, Federico, VP, et al. (2022) (26)  Hinkka, K, Karppi, SL, Pohjolainen, T, et al. (2007) (79)  ClinicalTrials.gov identifier: NCT04715581 (54)  Trialsearch.who.int identifier: ACTRN12620001173987 (63)  Serra-Prat, M, Terradellas, M, Lorenzo, I, et al. (2022) (55) | Pain | Participants asked to numerically rate overall pain, back pain, leg pain or chronic pain  Numeric Rating Scales and Visual Analogue Scales used |
| Geriatric Pain Measure | 1 | Ferrell, BA, Stein, WM, Beck, JC (2000) (155) | Pain | 24 items on a 2-point scale assessing geriatric pain |
| PROMIS Pain - Behaviour (v1.0) | 1 | HealthMeasures (2022) (97) | Pain | 20 items on a 5-point scale on behaviours that indicate to others that an individual is experiencing pain |
| PROMIS Pain - Interference (v1.1) | 1 | HealthMeasures (2022) (97) | Pain | 40 items on a 5-point scale on the consequences of pain to an individual's life |
| IPOS | 2 | Murtagh, FE, Ramsenthaler, C, Firth, A, et al. (2019) (156) | Palliative Care | 10 items, including symptoms and mental wellbeing |
| ICECAP supportive care measure (ICECAP-SCM) | 1 | Sutton, EJ, Coast, J (2014) (157) | Palliative Care | 7 items on outcomes that matter at end-of-life, with 4 levels each |
| IPOS - Views on Care | 1 | Addington-Hall, J, Hunt, K, Rowsell, A, et al. (2014) (158) | Palliative Care | 5 items assessing perceived impact of palliative healthcare |
| Adapted Control Preference Scale | 1 | Degner, LF, Sloan, JA, Venkatesh, P. (1997) (159) | Participation in Decision-Making | Scale of 1-5 describing varying levels of patient involvement in decision-making |
| Gothenburg Empowerment Scale | 1 | Acuña Mora, M, Raymaekers, K, Van Bulck, L, et al. (2022) (160) | Participation in Decision-Making | 15 items measuring 5 dimensions of patient empowerment: personal control, knowledge and understanding, identity, shared decision-making, and enabling others |
| Health Care Climate Questionnaire (HCCQ) | 1 | Czajkowska, Z, Wang, H, Hall, NC, et al. (2017) (161) | Participation in Decision-Making | 15 items on a 7-point scale, assessing patients' perspectives of their autonomy in healthcare |
| Community Health Activities Model Program for Seniors (CHAMPS) | 2 | Stewart, AL, Mills, KM, King, AC, et al. (2001) (162) | Physical Activity | 41 items, assessing frequency and duration of physical activity |
| International Physical Activity Questionnaire (IPAQ) | 3 | Craig, CL, Marshall, AL, Sjöström, M, et al. (2003) (163) | Physical Activity | Assesses physical activity level, including number of days and amount of time on each day spent on degrees of physical activity  Multiple versions exist, including:  Short (9-item) and Long (31-item) versions Can be administered by telephone or self Reference period of the "last 7 days", or a "usual week" |
| Beijing Longitudinal Study of Ageing physical activity questionnaire (BLSA PAQ) | 1 | Ma, L, Wang, J, Tang, Z, et al. (2018) (164) | Physical Activity | 4 items assessing gardening, walking, low-level and high-level exercise, each with 4 levels Currently exists only in Chinese |
| Godin-Shephard Leisure-Time Physical Activity Questionnaire | 1 | Godin, G (2011) (165) | Physical Activity | 3 items assessing frequency of strenuous exercise, moderate exercise and mild exercise Items are weighted by activity level to produce a score |
| Modified Baecke physical activity questionnaire | 1 | Voorrips, LE, Ravelli, AC, Dongelmans, PC, et al. (1991) (166) | Physical Activity | 3 sections of items on household, sport, and leisure activities Modified for use in older adults |
| Physical Activity Scale for the Elderly (PASE) | 1 | Washburn, RA, Smith, KW, Jette, AM, et al. (1993) (167) | Physical Activity | 10 items assessing self-reported participation in leisure, household and work activities |
| Ad-hoc Physical Activity Questionnaire | 1 | Franse, CB, van Grieken, A, Alhambra-Borrás, T, et al. (2018) (53) | Physical Activity | 1 item assessing whether a person engaged in activities that require low or moderate energy |
| Cognitive Failures Questionnaire (CFQ) | 1 | Broadbent, DE, Cooper, PF, FitzGerald, P, et al. (1982) (168) | Psychological Functioning | 25 items on a 5-point scale, assessing minor failures in attention, perception and memory |
| International Index of Erectile Function | 3 | Rosen, RC, Riley, A, Wagner, G, et al. (1997) (169) | Sexual Quality of Life | 15 items with 5-6 levels reporting male sexual functioning |
| Sexual Quality of Life-Female (SQOL-F) | 2 | Symonds, T, Boolell, M, Quirk, F. (2005) (170) | Sexual Quality of Life | 18 items measuring female sexual quality of life, with 6 levels each  SQOL-M also exists for males, but was not used |
| Female Sexual Function Index FSFI | 1 | Wiegel, M, Meston, C, Rosen, R (2005) (171) | Sexual Quality of Life | 19 items with 5-6 levels assessing female sexual functioning |
| Pittsburgh Sleep quality Index | 2 | Buysse, DJ, Reynolds, CF, 3rd, Monk, TH, et al. (1989) (172) | Sleep Quality | Assesses sleep quality in clinical populations  7 components: subjective sleep quality, sleep latency, sleep duration, habitual sleep efficiency, sleep disturbances, use of sleeping medications, and daytime dysfunction  Includes 5 items answered by roommate or partner if applicable |
| Insomnia Severity Index | 1 | Morin, CM, Belleville, G, Bélanger L, et al. (2011) (173) | Sleep Quality | 7 items on a 5-point scale assessing nature, severity and impact of insomnia |
| Sleep Quality Scale (Numeric) | 1 | ClinicalTrials.gov identifier: NCT04715581 (54) | Sleep Quality | Rating of sleep quality on an 11-point scale |
| Custom Social Activities/Participation | 2 | Fairhall, N, Aggar, C, Kurrle, SE, et al. (2008) (38) Gilmore, N, Kehoe, L, Bauer, J, et al. (2021) (61) | Social Activities | Variable between studies Participants asked to assess their social activity levels |
| Social Activity Scale (Linear Analogue) | 1 | Kotajarvi, BR, Schafer, MJ, Atkinson, EJ, et al. (2017) (20) | Social Activities | Participants asked to rate levels of social activity |
| PROMIS Satisfaction with Social Roles and Activities (v2.0) | 1 | HealthMeasures (2022) (97) | Social Activities | 44 items on a 5-point scale measuring satisfaction with social roles and activities |
| MOS Social Support Survey | 2 | Sherbourne CD, Stewart AL (1991) (174) | Social Support | 20 items, 19 of which are on a 5-point scale  Designed to assess support for people with chronic conditions |
| Multidimensional Scale of Perceived Social Support (MSPSS) | 1 | Zimet, GD, Dahlem, NW, Zimet, SG, et al. (1988) (175) | Social Support | 12 items on a 7-point scale measuring perceived social support  3 subscales: significant other, family, and friends |
| Personal Resource Questionnaire (PRQ85) | 1 | Weinert, C, Brandt, PA (1987) (176) | Social Support | Part 1 assesses perceived social support for 10 life situations  Part 2 consists of 25 items on a 7-point scale |
| Social Provisions Scale (SPS) | 1 | Cutrona, C, Russell, D (1983) (177) | Social Support | 24 items on a 4-point scale assessing perceived social support |
| Nijmegen Continuity Questionnaire | 1 | Uijen, AA, Schellevis, FG, van den Bosch WJ, et al. (2011) (178) | Transitional Care | Final version contains 28 items assessing how well different care providers know the patient and communicate information with each other |
| Reintegration into Normal Living Index | 1 | Wood-Dauphinee, S, Williams, JI (1987) (179) | Transitional Care | 11 items on a 10-point scale Assesses important domains for returning to community living, including mobility, self-care and social activities |
| Total number of usages of PROMs | 289 |  |  |  |
| Total number of unique PROMs | 112 |  |  |  |

There are 115 descriptions of instruments. However, the final tally of 112 unique instruments excludes 3 instruments that were used with both patients and carers: the Hospital Anxiety and Depression Scale (HADS), the Client Satisfaction Questionnaire (CSQ-8) and PROMIS Global Health, as described in the Methods section.

# Appendix 4 – Supplementary Fig. 1 Examples of Types of Rating Scales for Pain


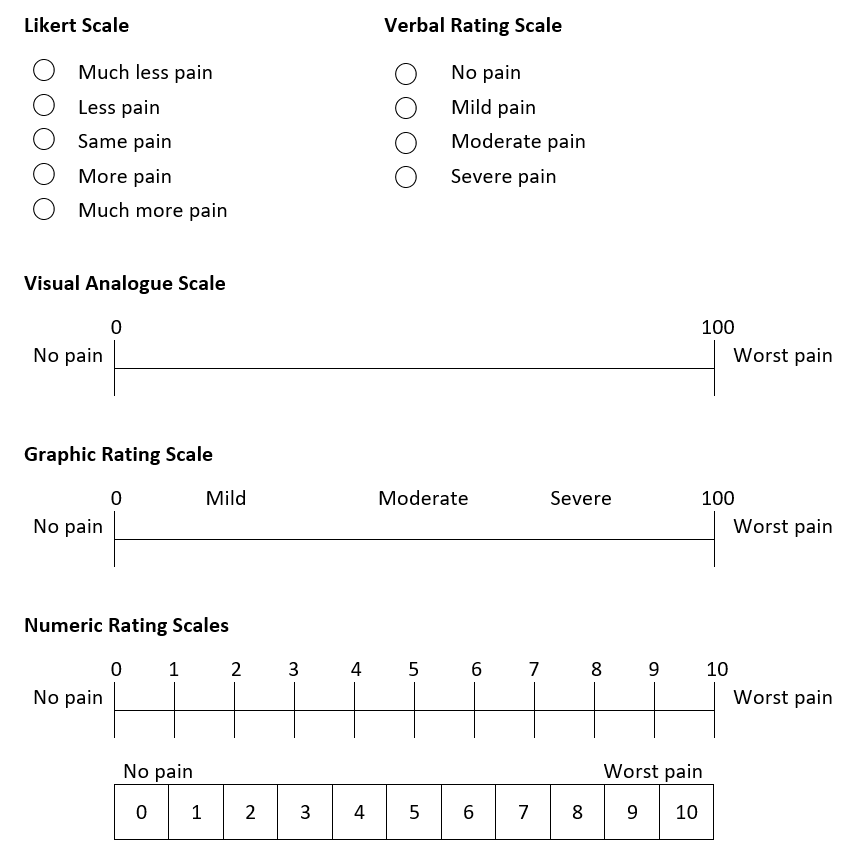


# Appendix 5 – Supplementary Reference List

1. Carli F, Bousquet-Dion G, Awasthi R, Elsherbini N, Liberman S, Boutros M, et al. Effect of Multimodal Prehabilitation vs Postoperative Rehabilitation on 30-Day Postoperative Complications for Frail Patients Undergoing Resection of Colorectal Cancer: a Randomized Clinical Trial. JAMA surgery. 2020;155(3):233‐42.

2. Justice JN, Nambiar AM, Tchkonia T, LeBrasseur NK, Pascual R, Hashmi SK, et al. Senolytics in idiopathic pulmonary fibrosis: Results from a first-in-human, open-label, pilot study. EBioMedicine. 2019;40:554-63.

3. Moye J, Driver JA, Owsiany MT, Chen LQ, Whitley JC, Auguste EJ, et al. Assessing What Matters Most in Older Adults With Multicomplexity. Gerontologist. 2022;62(4):e224-e34.

4. Kusunose M, Sanda R, Mori M, Narita A, Nishimura K. Are frailty and patient-reported outcomes independent in subjects with asthma? A cross-sectional observational study. Clin Respir J. 2021;15(2):216-24.

5. Frantzen AT, Eide LSP, Fridlund B, Haaverstad R, Hufthammer KO, Kuiper KKJ, et al. Frailty Status and Patient-Reported Outcomes in Octogenarians Following Transcatheter or Surgical Aortic Valve Replacement. Heart Lung Circ. 2021;30(8):1221-31.

6. Knight J, Ayyash K, Colling K, Dhesi J, Ewan V, Danjoux G, et al. A cohort study investigating the relationship between patient reported outcome measures and pre-operative frailty in patients with operable, non-palliative colorectal cancer. BMC Geriatr. 2020;20(1):311.

7. Liu Z, Han L, Gahbauer EA, Allore HG, Gill TM. Joint Trajectories of Cognition and Frailty and Associated Burden of Patient-Reported Outcomes. J Am Med Dir Assoc. 2018;19(4):304-9.e2.

8. Milne B, Lucas de Carvalho J, Ayis S, Chaubey S, Khan H, Kunst G. Frailty and perioperative patient-reported disability in patients undergoing cardiac surgery: a pilot study. Br J Anaesth. 2022;128(6):949-58.

9. Tapper EB, Baki J, Parikh ND, Lok AS. Frailty, Psychoactive Medications, and Cognitive Dysfunction Are Associated With Poor Patient-Reported Outcomes in Cirrhosis. Hepatology. 2019;69(4):1676-85.

10. Martillo MA, Dangayach NS, Tabacof L, Spielman LA, Dams-O'Connor K, Chan CC, et al. Postintensive Care Syndrome in Survivors of Critical Illness Related to Coronavirus Disease 2019: Cohort Study From a New York City Critical Care Recovery Clinic. Crit Care Med. 2021;49(9):1427-38.

11. Lieber SB, Nahid M, Paget S, Berman JR, Barbhaiya M, Sammaritano LR, et al. Evaluation of a Patient-reported Frailty Tool in Women With Systemic Lupus Erythematosus. J Rheumatol. 2022;49(1):60-7.

12. Tejiram S, Galet C, Cartwright J, Hatcher VH, Skeete DA, Cocanour C, et al. Association of Patient Reported Global Health Measures with Frailty Scores in Acutely Injured Older Adults. J Frailty Aging. 2022;11(1):67-73.

13. Geense WW, Zegers M, Peters MAA, Ewalds E, Simons KS, Vermeulen H, et al. New Physical, Mental, and Cognitive Problems 1 Year after ICU Admission: A Prospective Multicenter Study. Am J Respir Crit Care Med. 2021;203(12):1512-21.

14. Prichard RA, Zhao FL, McDonagh J, Goodall S, Davidson PM, Newton PJ, et al. Discrepancies between proxy estimates and patient reported, health related, quality of life: minding the gap between patient and clinician perceptions in heart failure. Qual Life Res. 2021;30(4):1049-59.

15. Tapper EB, Kenney B, Nikirk S, Levine DA, Waljee AK. Animal Naming Test Is Associated With Poor Patient-Reported Outcomes and Frailty in People With and Without Cirrhosis: A Prospective Cohort Study. Clin Transl Gastroenterol. 2022;13(1):e00447.

16. Van Bulck L, Kovacs AH, Goossens E, Luyckx K, Zaidi A, Wang JK, et al. Rationale, design and methodology of APPROACH-IS II: International study of patient-reported outcomes and frailty phenotyping in adults with congenital heart disease. Int J Cardiol. 2022;363:30-9.

17. Jonsson H, Piscator E, Israelsson J, Lilja G, Djärv T. Is frailty associated with long-term survival, neurological function and patient-reported outcomes after in-hospital cardiac arrest? - A Swedish cohort study. Resuscitation. 2022;179:233-42.

18. Holeman TA, Peacock J, Beckstrom JL, Brooke BS. Patient-Surgeon Agreement in Assessment of Frailty, Physical Function, & Social Activity. J Surg Res. 2020;256:368-73.

19. Reid DBC, Daniels AH, Ailon T, Miller E, Sciubba DM, Smith JS, et al. Frailty and Health-Related Quality of Life Improvement Following Adult Spinal Deformity Surgery. World Neurosurg. 2018;112:e548-e54.

20. Kotajarvi BR, Schafer MJ, Atkinson EJ, Traynor MM, Bruce CJ, Greason KL, et al. The Impact of Frailty on Patient-Centered Outcomes Following Aortic Valve Replacement. J Gerontol A Biol Sci Med Sci. 2017;72(7):917-21.

21. Hanlon P, Morton F, Siebert S, Jani BD, Nicholl BI, Lewsey J, et al. Frailty in rheumatoidrmdopen-2021-002111 arthritis and its relationship with disease activity, hospitalisation and mortality: a longitudinal analysis of the Scottish Early Rheumatoid Arthritis cohort and UK Biobank. RMD Open. 2022;8(1).

22. Ponjee GHM, van de Meerendonk H, Janssen MJA, Karapinar-Çarkit F. The effect of an inpatient geriatric stewardship on drug-related problems reported by patients after discharge. Int J Clin Pharm. 2021;43(1):191-202.

23. Boumans R, van Meulen F, Hindriks K, Neerincx M, Olde Rikkert MGM. Robot for health data acquisition among older adults: a pilot randomised controlled cross-over trial. BMJ Qual Saf. 2019;28(10):793-9.

24. Zisiopoulou M, Berkowitsch A, Seppelt P, Zeiher AM, Vasa-Nicotera M. A Novel Method to Predict Mortality and Length of Stay after Transfemoral Transcatheter Aortic Valve Implantation. Medicina (Kaunas). 2021;57(12).

25. Boucher V, Lamontagne ME, Lee J, Carmichael PH, Déry J, Émond M. Acceptability of older patients' self-assessment in the Emergency Department (ACCEPTED)-a randomised cross-over pilot trial. Age Ageing. 2019;48(6):875-80.

26. Zavras AG, Sullivan TB, Federico VP, Nolte MT, Munim MA, Phillips FM, et al. Preoperative Multifidus Muscle Quality is Associated With Patient Reported Outcomes After Lateral Lumbar Interbody Fusion. Global Spine J. 2022:21925682221120400.

27. Nicholson C, Davies JM, George R, Smith B, Pace V, Harris L, et al. What are the main palliative care symptoms and concerns of older people with multimorbidity?-a comparative cross-sectional study using routinely collected Phase of Illness, Australia-modified Karnofsky Performance Status and Integrated Palliative Care Outcome Scale data. Ann Palliat Med. 2018;7(Suppl 3):S164-s75.

28. McIsaac DI, Taljaard M, Bryson GL, Beaule PE, Gagne S, Hamilton G, et al. Comparative assessment of two frailty instruments for risk-stratification in elderly surgical patients: study protocol for a prospective cohort study. BMC Anesthesiol. 2016;16(1):111.

29. Murata S, Tsutsui S, Hashizume H, Minamide A, Nakagawa Y, Iwasaki H, et al. Importance of physiological age in determining indications for adult spinal deformity surgery in patients over 75 years of age: a propensity score matching analysis. Eur Spine J. 2022.

30. Quinten C, Kenis C, Hamaker M, Coolbrandt A, Brouwers B, Dal Lago L, et al. The effect of adjuvant chemotherapy on symptom burden and quality of life over time; a preliminary prospective observational study using individual data of patients aged ≥70 with early stage invasive breast cancer. J Geriatr Oncol. 2018;9(2):152-62.

31. Efficace F, Boccadoro M, Palumbo A, Petrucci MT, Cottone F, Cannella L, et al. A prospective observational study to assess clinical decision-making, prognosis, quality of life and satisfaction with care in patients with relapsed/refractory multiple myeloma: the CLARITY study protocol. Health Qual Life Outcomes. 2018;16(1):127.

32. Abdelaziz HK, Hashmi I, Taylor R, Debski M, Hasan R, Rajathurai T, et al. Quality of Life Assessment in Patients Undergoing Trans-Catheter Aortic Valve Implantation Using MacNew Questionnaire. Am J Cardiol. 2022;164:103-10.

33. Zisiopoulou M, Berkowitsch A, Neuber R, Gouveris H, Fichtlscherer S, Walther T, et al. Personalized Preoperative Prediction of the Length of Hospital Stay after TAVI Using a Dedicated Decision Tree Algorithm. J Pers Med. 2022;12(3).

34. Orange ST, Hallsworth K, Brown MC, Reeves HL. The feasibility and acceptability of a home-based, virtual exercise intervention for older patients with hepatocellular carcinoma: protocol for a non-randomised feasibility study (TELEX-Liver Cancer). Pilot Feasibility Stud. 2022;8(1):113.

35. Bally ELS, van Grieken A, Ye L, Ferrando M, Fernández-Salido M, Dix R, et al. 'Value-based methodology for person-centred, integrated care supported by Information and Communication Technologies' (ValueCare) for older people in Europe: study protocol for a pre-post controlled trial. BMC Geriatr. 2022;22(1):680.

36. Ekdahl AW, Wirehn A-B, Alwin J, Jaarsma T, Unosson M, Husberg M, et al. Costs and Effects of an Ambulatory Geriatric Unit (the AGe-FIT Study): A Randomized Controlled Trial. Journal of the American Medical Directors Association. 2015;16(6):497-503.

37. Eckerblad J, Theander K, Ekdahl AW, Jaarsma T. Symptom trajectory and symptom burden in older people with multimorbidity, secondary outcome from the RCT AGe-FIT study. J Adv Nurs. 2016;72(11):2773-83.

38. Fairhall N, Aggar C, Kurrle SE, Sherrington C, Lord S, Lockwood K, et al. Frailty Intervention Trial (FIT). BMC Geriatr. 2008;8:27.

39. Aggar C, Ronaldson S, Cameron ID. Reactions to caregiving during an intervention targeting frailty in community living older people. BMC Geriatr. 2012;12:66.

40. Mohd Suffian NI, Adznam SN, Abu Saad H, Chan YM, Ibrahim Z, Omar N, et al. Frailty Intervention through Nutrition Education and Exercise (FINE). A Health Promotion Intervention to Prevent Frailty and Improve Frailty Status among Pre-Frail Elderly-A Study Protocol of a Cluster Randomized Controlled Trial. Nutrients. 2020;12(9).

41. Jang IY, Jung HW, Park H, Lee CK, Yu SS, Lee YS, et al. A multicomponent frailty intervention for socioeconomically vulnerable older adults: a designed-delay study. Clin Interv Aging. 2018;13:1799-814.

42. Anderson BM, Dutton M, Day E, Jackson TA, Ferro CJ, Sharif A. Frailty Intervention Trial iN End-Stage patientS on haemodialysis (FITNESS): study protocol for a randomised controlled trial. Trials. 2018;19(1):457.

43. de Nooijer K, Pivodic L, Van Den Noortgate N, Pype P, Van den Block L. Timely short-term specialised palliative care service intervention for frail older people and their family carers in primary care: study protocol for a pilot randomised controlled trial. BMJ Open. 2021;11(1):e043663.

44. de Nooijer K, Van Den Noortgate N, Pype P, Van den Block L, Pivodic L. Palliative care symptoms, concerns and well-being of older people with frailty and complex care needs upon hospital discharge: a cross-sectional study. BMC palliative care. 2022;21(1):173.

45. Ma L, Zhang L, Sun F, Li Y, Tang Z. Frailty in Chinese older adults with hypertension: Prevalence, associated factors, and prediction for long-term mortality. J Clin Hypertens (Greenwich). 2018;20(11):1595-602.

46. Bleijenberg N, Drubbel I, Ten Dam VH, Numans ME, Schuurmans MJ, de Wit NJ. Proactive and integrated primary care for frail older people: design and methodological challenges of the Utrecht primary care PROactive frailty intervention trial (U-PROFIT). BMC Geriatr. 2012;12:16.

47. Chong E, Zhu B, Ng SHX, Tan H, Goh EF, Molina JC, et al. Emergency department interventions for frailty (EDIFY): improving functional outcomes in older persons at the emergency department through a multicomponent frailty intervention. Age Ageing. 2022;51(2).

48. Yu R, Tong C, Leung G, Woo J. Assessment of the validity and acceptability of the online FRAIL scale in identifying frailty among older people in community settings. Maturitas. 2021;145:18-23.

49. Liu LK, Lee WJ, Chen LY, Hwang AC, Lin MH, Peng LN, et al. Association between Frailty, Osteoporosis, Falls and Hip Fractures among Community-Dwelling People Aged 50 Years and Older in Taiwan: Results from I-Lan Longitudinal Aging Study. PLoS One. 2015;10(9):e0136968.

50. Liang CK, Lee WJ, Hwang AC, Lin CS, Chou MY, Peng LN, et al. Efficacy of Multidomain Intervention Against Physio-cognitive Decline Syndrome: A Cluster-randomized Trial. Arch Gerontol Geriatr. 2021;95:104392.

51. Negm AM, Kennedy CC, Ioannidis G, Gajic-Veljanoski O, Lee J, Thabane L, et al. Getting fit for hip and knee replacement: a protocol for the Fit-Joints pilot randomized controlled trial of a multi-modal intervention in frail patients with osteoarthritis. Pilot Feasibility Stud. 2018;4:127.

52. Block H, Annesley A, Lockwood K, Xu L, Cameron ID, Laver K, et al. Frailty in older people: Rehabilitation Treatment Research Examining Separate Settings (FORTRESS): protocol for a hybrid type II stepped wedge, cluster, randomised trial. BMC Geriatr. 2022;22(1):527.

53. Franse CB, van Grieken A, Alhambra-Borrás T, Valía-Cotanda E, van Staveren R, Rentoumis T, et al. The effectiveness of a coordinated preventive care approach for healthy ageing (UHCE) among older persons in five European cities: A pre-post controlled trial. Int J Nurs Stud. 2018;88:153-62.

54. Nct. Multicomponent Prehabilitation and Outcomes in Elderly Patients With Frailty. <https://clinicaltrialsgov/show/NCT04715581>. 2021.

55. Serra-Prat M, Terradellas M, Lorenzo I, Arús M, Burdoy E, Salietti A, et al. Effectiveness of a Weight-Loss Intervention in Preventing Frailty and Functional Decline in Community-Dwelling Obese Older People. A Randomized Controlled Trial. The Journal of frailty & aging. 2022;11(1):91‐9.

56. Actrn. Standardised assessment and management of frailty in elderly respiratory patients. <https://trialsearchwhoint/Trial2aspx?TrialID=ACTRN12612000678897>. 2012.

57. Kennedy CC, Novotny PJ, LeBrasseur NK, Wise RA, Sciurba FC, Benzo RP. Frailty and Clinical Outcomes in Chronic Obstructive Pulmonary Disease. Annals of the American Thoracic Society. 2019;16(2):217‐24.

58. Yousefi K, Ramdas KN, Ruiz JG, Walston J, Arai H, Volpi E, et al. The Design and Rationale of a Phase 2b, Randomized, Double-Blinded, and Placebo-Controlled Trial to Evaluate the Safety and Efficacy of Lomecel-B in Older Adults with Frailty. The Journal of frailty & aging. 2022;11(2):214‐23.

59. Isrctn. The Yorkshire & Humber community ageing research study. <https://trialsearchwhoint/Trial2aspx?TrialID=ISRCTN16588124>. 2015.

60. Nct. Effects of Long-term Intensive Home-based Physiotherapy on Older People With an Operated Hip Fracture or Frailty (RCT). <https://clinicaltrialsgov/show/NCT02305433>. 2014.

61. Gilmore N, Kehoe L, Bauer J, Xu H, Hall B, Wells M, et al. The Relationship Between Frailty and Emotional Health in Older Patients with Advanced Cancer. Oncologist. 2021;26(12):e2181-e91.

62. McIsaac DI, Fergusson DA, Khadaroo R, Meliambro A, Muscedere J, Gillis C, et al. PREPARE trial: a protocol for a multicentre randomised trial of frailty-focused preoperative exercise to decrease postoperative complication rates and disability scores. BMJ Open. 2022;12(8):e064165.

63. Actrn. Optimising outcomes for frail hospitalised older adults – nurse led volunteer support and pain assessment interventions: a cluster randomised control trial. <https://trialsearchwhoint/Trial2aspx?TrialID=ACTRN12620001173987>. 2020.

64. Hirai K, Homma T, Matsunaga T, Akimoto K, Yamamoto S, Suganuma H, et al. Usefulness of Ninjin'yoeito for Chronic Obstructive Pulmonary Disease Patients with Frailty. Journal of alternative and complementary medicine (New York, NY). 2020;26(8):750‐7.

65. Irct20201108049310N. Effect of lifestyle education based on pender model on frailty outcomes in community-dwelling older adults. <https://trialsearchwhoint/Trial2aspx?TrialID=IRCT20201108049310N1>. 2020.

66. Villareal DT, Banks M, Sinacore DR, Siener C, Klein S. Effect of weight loss and exercise on frailty in obese older adults. Archives of internal medicine. 2006;166(8):860‐6.

67. Young H, March D, Highton P, Graham-Brown M, Goodliffe S, Greenwood S, et al. Exercise interventions for people living with frailty and receiving haemodialysis: a mixed-methods randomised controlled feasibility study. Physiotherapy (united kingdom). 2021;113:e4‐e5.

68. Chan DC, Tsou HH, Yang RS, Tsauo JY, Chen CY, Hsiung CA, et al. A pilot randomized controlled trial to improve geriatric frailty. BMC geriatrics. 2012;12:58.

69. Griffin AC, O'Neill A, O'Connor M, Ryan D, Tierney A, Galvin R. Malnutrition among older adults presenting at an irish emergency department: prevalence and impact on patient outcomes. Clinical nutrition ESPEN. 2020;40:582‐3.

70. Gomes GCV, Simões MDS, Lin SM, Bacha JMR, Viveiro LAP, Varise EM, et al. Feasibility, safety, acceptability, and functional outcomes of playing Nintendo Wii Fit PlusTM for frail older adults: a randomized feasibility clinical trial. Maturitas. 2018;118:20‐8.

71. Tompkins BA, DiFede DL, Khan A, Landin AM, Schulman IH, Pujol MV, et al. Allogeneic Mesenchymal Stem Cells Ameliorate Aging Frailty: a Phase II Randomized, Double-Blind, Placebo-Controlled Clinical Trial. Journals of gerontology Series A, Biological sciences and medical sciences. 2017;72(11):1513‐22.

72. Actrn. A study assessing the impact of frailty on therapy in older people with blood cancers. <https://trialsearchwhoint/Trial2aspx?TrialID=ACTRN12620000271909>. 2020.

73. Applegate WB, Miller ST, Graney MJ, Elam JT, Burns R, Akins DE. A Randomized, Controlled Trial of a Geriatric Assessment Unit in a Community Rehabilitation Hospital. New England Journal of Medicine. 1990;322(22):1572-8.

74. HOGAN DB, FOX RA. A Prospective Controlled Trial of a Geriatric Consultation Team in an Acute-care Hospital. Age and Ageing. 1990;19(2):107-13.

75. Winograd CH, Gerety MB, Lai NA. A Negative Trial of Inpatient Geriatric Consultation: Lessons Learned and Recommendations for Future Research. Archives of Internal Medicine. 1993;153(17):2017-23.

76. Karppi P. Effects of a geriatric inpatient unit on elderly home-care patients: A controlled trial. Aging Clinical and Experimental Research. 1995;7(3):207-11.

77. Cohen HJ, Feussner JR, Weinberger M, Carnes M, Hamdy RC, Hsieh F, et al. A Controlled Trial of Inpatient and Outpatient Geriatric Evaluation and Management. New England Journal of Medicine. 2002;346(12):905-12.

78. Saltvedt I, Jordhøy M, Opdahl Mo ES, Fayers P, Kaasa S, Sletvold O. Randomised Trial of In-Hospital Geriatric Intervention: Impact on Function and Morale. Gerontology. 2006;52(4):223-30.

79. Hinkka K, Karppi SL, Pohjolainen T, Rantanen T, Puukka P, Tilvis R. Network-based geriatric rehabilitation for frail elderly people: feasibility and effects on subjective health and pain at one year. J Rehabil Med. 2007;39(6):473-8.

80. Ollonqvist K, Aaltonen T, Karppi S-L, Hinkka K, Pöntinen S. Network-based rehabilitation increases formal support of frail elderly home-dwelling persons in Finland: randomised controlled trial. Health & Social Care in the Community. 2008;16(2):115-25.

81. Kehusmaa S, Autti-Rämö I, Valaste M, Hinkka K, Rissanen P. Economic evaluation of a geriatric rehabilitation programme: a randomized controlled trial. J Rehabil Med. 2010;42(10):949-55.

82. Kircher TTJ, Wormstall H, Müller PH, Schwärzler F, Buchkremer G, Wild K, et al. A randomised trial of a geriatric evaluation and management consultation services in frail hospitalised patients. Age and Ageing. 2007;36(1):36-42.

83. Torres-Sánchez I, Valenza MC, Cabrera-Martos I, López-Torres I, Benítez-Feliponi Á, Conde-Valero A. Effects of an Exercise Intervention in Frail Older Patients with Chronic Obstructive Pulmonary Disease Hospitalized due to an Exacerbation: A Randomized Controlled Trial. COPD: Journal of Chronic Obstructive Pulmonary Disease. 2017;14(1):37-42.

84. Bernabei R, Landi F, Gambassi G, Sgadari A, Zuccala G, Mor V, et al. Randomised trial of impact of model of integrated care and case management for older people living in the community. Bmj. 1998;316(7141):1348-51.

85. Shannon GR, Wilber KH, Allen D. Reductions in Costly Healthcare Service Utilization: Findings from the Care Advocate Program. Journal of the American Geriatrics Society. 2006;54(7):1102-7.

86. Markle-Reid M, Weir R, Browne G, Roberts J, Gafni A, Henderson S. Health promotion for frail older home care clients. Journal of Advanced Nursing. 2006;54(3):381-95.

87. Béland F, Bergman H, Lebel P, Clarfield AM, Tousignant P, Contandriopoulos A-P, et al. A System of Integrated Care for Older Persons With Disabilities in Canada: Results From a Randomized Controlled Trial. The Journals of Gerontology: Series A. 2006;61(4):367-73.

88. Gagnon AJ, Schein C, McVey L, Bergman H. Randomized controlled trial of nurse case management of frail older people. J Am Geriatr Soc. 1999;47(9):1118-24.

89. Rockwood K, Stadnyk K, Carver D, MacPherson KM, Beanlands HE, Powell C, et al. A clinimetric evaluation of specialized geriatric care for rural dwelling, frail older people. J Am Geriatr Soc. 2000;48(9):1080-5.

90. Newcomer R, Maravilla V, Faculjak P, Graves MT. Outcomes of Preventive Case Management Among High-Risk Elderly in Three Medical Groups:A Randomized Clinical Trial. Evaluation & the Health Professions. 2004;27(4):323-48.

91. Leveille SG, Wagner EH, Davis C, Grothaus L, Wallace J, LoGerfo M, et al. Preventing Disability and Managing Chronic Illness in Frail Older Adults: A Randomized Trial of a Community-Based Partnership with Primary Care. Journal of the American Geriatrics Society. 1998;46(10):1191-8.

92. Ambrosius WT, Sink KM, Foy CG, Berlowitz DR, Cheung AK, Cushman WC, et al. The design and rationale of a multicenter clinical trial comparing two strategies for control of systolic blood pressure: the Systolic Blood Pressure Intervention Trial (SPRINT). Clin Trials. 2014;11(5):532-46.

93. Actrn. Does provision of computerised cognitive training for an extended period of time in older hospitalised patients help in improving their frailty status? <https://trialsearchwhoint/Trial2aspx?TrialID=ACTRN12620000607976>. 2020.

94. Mahoney FI, Barthel DW. Functional evaluation: The Barthel Index: A simple index of independence useful in scoring improvement in the rehabilitation of the chronically ill. Maryland State Medical Journal. 1965;14:61-5.

95. Lawton MP, Brody EM. Assessment of older people: self-maintaining and instrumental activities of daily living. Gerontologist. 1969;9(3):179-86.

96. Katz S, Downs TD, Cash HR, Grotz RC. Progress in development of the index of ADL. Gerontologist. 1970;10(1):20-30.

97. HealthMeasures. Intro to PROMIS [Available from: <www.healthmeasures.net/explore-measurement-systems/promis/intro-to-promis>.

98. Fillenbaum G. Multidimensional functional assessment of older adults: The Duke Older Americans Resources and Services Procedures. Hillsdale, N.J.: Erlbaum; 1988 (updated 2005, available only from Center for the Study of Aging and Human Development, Duke University Medical Center, Durham, NC 27710).

99. World Health Organisation WHO disability assessment schedule 2.0 (WHODAS 2.0) 2012 [cited 2022 16 Dec]. Available from: <https://www.who.int/classifications/international-classification-of-functioning-disability-and-health/who-disability-assessment-schedule>.

100. Hlatky MA, Boineau RE, Higginbotham MB, Lee KL, Mark DB, Califf RM, et al. A brief self-administered questionnaire to determine functional capacity (The Duke Activity Status Index). American Journal of Cardiology. 1989;64(10):651-4.

101. Suurmeijer TP, Doeglas DM, Moum T, Briançon S, Krol B, Sanderman R, et al. The Groningen Activity Restriction Scale for measuring disability: its utility in international comparisons. Am J Public Health. 1994;84(8):1270-3.

102. Hawley DJ, Wolfe F. Sensitivity to change of the Health Assessment Questionnaire (HAQ) and other clinical and health status measures in rheumatoid arthritis results of short-term clinical trials and observational studies versus long-term observational studies. Arthritis & Rheumatism. 1992;5(3):130-6.

103. Nouri F, Lincoln N. An extended activities of daily living scale for stroke patients. Clinical Rehabilitation. 1987;1(4):301-5.

104. Malmstrom TK, Morley JE. SARC-F: a simple questionnaire to rapidly diagnose sarcopenia. J Am Med Dir Assoc. 2013;14(8):531-2.

105. Nagi SZ. An epidemiology of disability among adults in the United States. Milbank Mem Fund Q Health Soc. 1976;54(4):439-67.

106. Katz P, Morris A, Trupin L, Yazdany J, Yelin E. Disability in valued life activities among individuals with systemic lupus erythematosus. Arthritis Rheum. 2008;59(4):465-73.

107. Peel C, Sawyer Baker P, Roth DL, Brown CJ, Brodner EV, Allman RM. Assessing mobility in older adults: the UAB Study of Aging Life-Space Assessment. Phys Ther. 2005;85(10):1008-119.

108. Netten A, Burge P, Malley J, Potoglou D, Towers AM, Brazier J, et al. Outcomes of social care for adults: developing a preference-weighted measure. 2012;16:16.

109. Zarit SH, Reever KE, Bach-Peterson J. Relatives of the impaired elderly: correlates of feelings of burden. Gerontologist. 1980;20(6):649-55.

110. Bouwmans C, Krol M, Severens H, Koopmanschap M, Brouwer W, Hakkaart-van Roijen L. The iMTA Productivity Cost Questionnaire: A Standardized Instrument for Measuring and Valuing Health-Related Productivity Losses. Value Health. 2015;18(6):753-8.

111. Petrinec A, Burant C, Douglas S. Caregiver reaction assessment: psychometric properties in caregivers of advanced cancer patients. Psychooncology. 2017;26(6):862-5.

112. Brouwer WB, van Exel NJ, van Gorp B, Redekop WK. The CarerQol instrument: a new instrument to measure care-related quality of life of informal caregivers for use in economic evaluations. Qual Life Res. 2006;15(6):1005-21.

113. Cooper B, Kinsella GJ, Picton C. Development and initial validation of a family appraisal of caregiving questionnaire for palliative care. Psychooncology. 2006;15(7):613-22.

114. Larsen DL, Attkisson CC, Hargreaves WA, Nguyen TD. Assessment of client/patient satisfaction: development of a general scale. Eval Program Plann. 1979;2(3):197-207.

115. Krevers B, Milberg A. The sense of security in care--Relatives' Evaluation instrument: its development and presentation. J Pain Symptom Manage. 2015;49(3):586-94.

116. Zigmond AS, Snaith RP. The hospital anxiety and depression scale. Acta Psychiatr Scand. 1983;67(6):361-70.

117. Kristensen TS, Borritz M, Villadsen E, Christensen KB. The Copenhagen Burnout Inventory: A new tool for the assessment of burnout. Work & Stress. 2005;19(3):192-207.

118. Weiss DJ, Dawis RV, England GW. Manual for the Minnesota Satisfaction Questionnaire. Minnesota Studies in Vocational Rehabilitation. 1967;22:120-.

119. Rafferty AM, Philippou J, Fitzpatrick JM, Pike G, Ball J. Development and testing of the ‘Culture of Care Barometer’ (CoCB) in healthcare organisations: a mixed methods study. BMJ Open. 2017;7(8):e016677.

120. Vercoulen JH, Swanink CM, Fennis JF, Galama JM, van der Meer JW, Bleijenberg G. Dimensional assessment of chronic fatigue syndrome. J Psychosom Res. 1994;38(5):383-92.

121. Smets EM, Garssen B, Bonke B, De Haes JC. The Multidimensional Fatigue Inventory (MFI) psychometric qualities of an instrument to assess fatigue. J Psychosom Res. 1995;39(3):315-25.

122. Valko PO, Bassetti CL, Bloch KE, Held U, Baumann CR. Validation of the fatigue severity scale in a Swiss cohort. Sleep. 2008;31(11):1601-7.

123. Webster K, Cella D, Yost K. The F unctional A ssessment of C hronic I llness T herapy (FACIT) Measurement System: properties, applications, and interpretation. Health and Quality of Life Outcomes. 2003;1(1):79.

124. Glynn NW, Santanasto AJ, Simonsick EM, Boudreau RM, Beach SR, Schulz R, et al. The Pittsburgh Fatigability scale for older adults: development and validation. J Am Geriatr Soc. 2015;63(1):130-5.

125. Krevers B, Milberg A. The instrument 'sense of security in care--patients' evaluation': its development and presentation. Psychooncology. 2014;23(8):914-20.

126. Ware JE, Jr., Sherbourne CD. The MOS 36-item short-form health survey (SF-36). I. Conceptual framework and item selection. Med Care. 1992;30(6):473-83.

127. Rabin R, de Charro F. EQ-5D: a measure of health status from the EuroQol Group. Ann Med. 2001;33(5):337-43.

128. Hays RD, Sherbourne CD, Mazel RM. The RAND 36-Item Health Survey 1.0. Health Econ. 1993;2(3):217-27.

129. Sintonen H. The 15D instrument of health-related quality of life: properties and applications. Ann Med. 2001;33(5):328-36.

130. Hawthorne G, Richardson J, Osborne R. The Assessment of Quality of Life (AQoL) instrument: a psychometric measure of health-related quality of life. Qual Life Res. 1999;8(3):209-24.

131. World Health O. The World Health Organization quality of life (WHOQOL) - BREF. Geneva: World Health Organization; 2004 2004. Contract No.: WHO/HIS/HSI Rev.2012.02.

132. Portenoy RK, Thaler HT, Kornblith AB, McCarthy Lepore J, Friedlander-Klar H, Kiyasu E, et al. The Memorial Symptom Assessment Scale: an instrument for the evaluation of symptom prevalence, characteristics and distress. European Journal of Cancer. 1994;30(9):1326-36.

133. van Oyen H, Van der Heyden J, Perenboom R, Jagger C. Monitoring population disability: evaluation of a new Global Activity Limitation Indicator (GALI). Soz Praventivmed. 2006;51(3):153-61.

134. Hyland ME, Sodergren SC. Development of a new type of global quality of life scale, and comparison of performance and preference for 12 global scales. Qual Life Res. 1996;5(5):469-80.

135. International Wellbeing Group Personal Wellbeing Index Melbourne: Australian Centre on Quality of Life, Deakin University; 2013 [5th Edition:[Available from: <http://www.acqol.com.au/instruments#measures>.

136. De Jong Gierveld J, Van Tilburg T. A 6-Item Scale for Overall, Emotional, and Social Loneliness: Confirmatory Tests on Survey Data. Research on Aging. 2006;28:582-98.

137. Hughes ME, Waite LJ, Hawkley LC, Cacioppo JT. A Short Scale for Measuring Loneliness in Large Surveys: Results From Two Population-Based Studies. Res Aging. 2004;26(6):655-72.

138. Barenholtz Levy H. Self-administered medication-risk questionnaire in an elderly population. Ann Pharmacother. 2003;37(7-8):982-7.

139. Willeboordse F, Grundeken LH, van den Eijkel LP, Schellevis FG, Elders PJ, Hugtenburg JG. Information on actual medication use and drug-related problems in older patients: questionnaire or interview? Int J Clin Pharm. 2016;38(2):380-7.

140. Yardley L, Beyer N, Hauer K, Kempen G, Piot-Ziegler C, Todd C. Development and initial validation of the Falls Efficacy Scale-International (FES-I). Age Ageing. 2005;34(6):614-9.

141. Kroll T, Kehn M, Ho PS, Groah S. The SCI Exercise Self-Efficacy Scale (ESES): development and psychometric properties. Int J Behav Nutr Phys Act. 2007;4:34.

142. Powell LE, Myers AM. The Activities-specific Balance Confidence (ABC) Scale. J Gerontol A Biol Sci Med Sci. 1995;50a(1):M28-34.

143. Yesavage JA, Brink TL, Rose TL, Lum O, Huang V, Adey M, et al. Development and validation of a geriatric depression screening scale: a preliminary report. J Psychiatr Res. 1982;17(1):37-49.

144. Radloff LS. The CES-D Scale: A self-report depression scale for research in the general population. Applied Psychological Measurement. 1977;1:385-401.

145. Kroenke K, Spitzer RL, Williams JB. The PHQ-9: validity of a brief depression severity measure. J Gen Intern Med. 2001;16(9):606-13.

146. Spitzer RL, Kroenke K, Williams JBW, Löwe B. A Brief Measure for Assessing Generalized Anxiety Disorder: The GAD-7. Archives of Internal Medicine. 2006;166(10):1092-7.

147. Parker G, Hilton T, Bains J, Hadzi-Pavlovic D. Cognitive-based measures screening for depression in the medically ill: the DMI-10 and the DMI-18. Acta Psychiatr Scand. 2002;105(6):419-26.

148. Spitzer RL, Williams JB, Kroenke K, Linzer M, deGruy FV, 3rd, Hahn SR, et al. Utility of a new procedure for diagnosing mental disorders in primary care. The PRIME-MD 1000 study. Jama. 1994;272(22):1749-56.

149. ZUNG WWK. A Self-Rating Depression Scale. Archives of General Psychiatry. 1965;12(1):63-70.

150. Lawton MP. The Philadelphia Geriatric Center Morale Scale: a revision. J Gerontol. 1975;30(1):85-9.

151. Wagnild GM, Young HM. Development and psychometric evaluation of the Resilience Scale. J Nurs Meas. 1993;1(2):165-78.

152. Molina Y, Choi SW, Cella D, Rao D. The stigma scale for chronic illnesses 8-item version (SSCI-8): development, validation and use across neurological conditions. Int J Behav Med. 2013;20(3):450-60.

153. Goal attainment scaling: Applications, theory, and measurement. Kiresuk TJ, Smith A, Cardillo JE, editors. Hillsdale, NJ, US: Lawrence Erlbaum Associates, Inc; 1994. xviii, 308-xviii, p.

154. Oris L, Luyckx K, Rassart J, Goubert L, Goossens E, Apers S, et al. Illness Identity in Adults with a Chronic Illness. J Clin Psychol Med Settings. 2018;25(4):429-40.

155. Ferrell BA, Stein WM, Beck JC. The Geriatric Pain Measure: validity, reliability and factor analysis. J Am Geriatr Soc. 2000;48(12):1669-73.

156. Murtagh FE, Ramsenthaler C, Firth A, Groeneveld EI, Lovell N, Simon ST, et al. A brief, patient- and proxy-reported outcome measure in advanced illness: Validity, reliability and responsiveness of the Integrated Palliative care Outcome Scale (IPOS). Palliat Med. 2019;33(8):1045-57.

157. Sutton EJ, Coast J. Development of a supportive care measure for economic evaluation of end-of-life care using qualitative methods. Palliat Med. 2014;28(2):151-7.

158. Addington-Hall J, Hunt K, Rowsell A, Heal R, Hansford P, Monroe B, et al. Development and initial validation of a new outcome measure for hospice and palliative care: the St Christopher's Index of Patient Priorities (SKIPP). BMJ Support Palliat Care. 2014;4(2):175-81.

159. Degner LF, Sloan JA, Venkatesh P. The Control Preferences Scale. Can J Nurs Res. 1997;29(3):21-43.

160. Acuña Mora M, Raymaekers K, Van Bulck L, Goossens E, Luyckx K, Kovacs AH, et al. Gothenburg Empowerment Scale (GES): psychometric properties and measurement invariance in adults with congenital heart disease from Belgium, Norway and South Korea. Health and Quality of Life Outcomes. 2022;20(1):145.

161. Czajkowska Z, Wang H, Hall NC, Sewitch M, Körner A. Validation of the English and French versions of the Brief Health Care Climate Questionnaire. Health Psychol Open. 2017;4(2):2055102917730675.

162. Stewart AL, Mills KM, King AC, Haskell WL, Gillis D, Ritter PL. CHAMPS physical activity questionnaire for older adults: outcomes for interventions. Med Sci Sports Exerc. 2001;33(7):1126-41.

163. CRAIG CL, MARSHALL AL, SJÖSTRÖM M, BAUMAN AE, BOOTH ML, AINSWORTH BE, et al. International Physical Activity Questionnaire: 12-Country Reliability and Validity. Medicine & Science in Sports & Exercise. 2003;35(8):1381-95.

164. Ma L, Wang J, Tang Z, Chan P. Simple Physical Activity Index Predicts Prognosis in Older Adults: Beijing Longitudinal Study of Aging. J Nutr Health Aging. 2018;22(7):854-60.

165. Godin G. The Godin-Shephard Leisure-Time Physical Activity Questionnaire. The Health & Fitness Journal of Canada. 2011;4(1):18-22.

166. Voorrips LE, Ravelli AC, Dongelmans PC, Deurenberg P, Van Staveren WA. A physical activity questionnaire for the elderly. Med Sci Sports Exerc. 1991;23(8):974-9.

167. Washburn RA, Smith KW, Jette AM, Janney CA. The Physical Activity Scale for the Elderly (PASE): development and evaluation. J Clin Epidemiol. 1993;46(2):153-62.

168. Broadbent DE, Cooper PF, FitzGerald P, Parkes KR. The Cognitive Failures Questionnaire (CFQ) and its correlates. British Journal of Clinical Psychology. 1982;21:1-16.

169. Rosen RC, Riley A, Wagner G, Osterloh IH, Kirkpatrick J, Mishra A. The international index of erectile function (IIEF): a multidimensional scale for assessment of erectile dysfunction. Urology. 1997;49(6):822-30.

170. Symonds T, Boolell M, Quirk F. Development of a questionnaire on sexual quality of life in women. J Sex Marital Ther. 2005;31(5):385-97.

171. Wiegel M, Meston C, Rosen R. The female sexual function index (FSFI): cross-validation and development of clinical cutoff scores. J Sex Marital Ther. 2005;31(1):1-20.

172. Buysse DJ, Reynolds CF, 3rd, Monk TH, Berman SR, Kupfer DJ. The Pittsburgh Sleep Quality Index: a new instrument for psychiatric practice and research. Psychiatry Res. 1989;28(2):193-213.

173. Morin CM, Belleville G, Bélanger L, Ivers H. The Insomnia Severity Index: psychometric indicators to detect insomnia cases and evaluate treatment response. Sleep. 2011;34(5):601-8.

174. Sherbourne CD, Stewart AL. The MOS social support survey. Soc Sci Med. 1991;32(6):705-14.

175. Zimet GD, Dahlem NW, Zimet SG, Farley GK. The Multidimensional Scale of Perceived Social Support. Journal of Personality Assessment. 1988;52:30-41.

176. Weinert C, Brandt PA. Measuring social support with the Personal Resource Questionnaire. West J Nurs Res. 1987;9(4):589-602.

177. Cutrona C, Russell D. The Provisions of Social Relationships and Adaptation to Stress. 11983. p. 37-67.

178. Uijen AA, Schellevis FG, van den Bosch WJ, Mokkink HG, van Weel C, Schers HJ. Nijmegen Continuity Questionnaire: development and testing of a questionnaire that measures continuity of care. J Clin Epidemiol. 2011;64(12):1391-9.

179. Wood-Dauphinee S, Williams JI. Reintegration to Normal Living as a proxy to quality of life. J Chronic Dis. 1987;40(6):491-502.
